# Viral Distribution of Wild Boar Exposed to Low (Vaccine Candidate) and High Virulence African Swine Fever Virus Isolates: Immunohistochemical Characterization

**DOI:** 10.1155/tbed/4258247

**Published:** 2025-12-09

**Authors:** Néstor Porras, Antonio Rodríguez-Bertos, Sandra Barroso-Arévalo, Aleksandra Kosowska, Marta Díaz de Frutos, Javier M. De Pablo-Moreno, Mónica Sánchez-Segovia, Jose Ángel Barasona

**Affiliations:** ^1^ VISAVET Health Surveillance Centre, Complutense University of Madrid, Madrid, Spain, ucm.es; ^2^ Department of Internal Medicine and Animal Surgery, Faculty of Veterinary Medicine, Complutense University of Madrid, Madrid, Spain, ucm.es; ^3^ SaBio, Institute for Game and Wildlife Research (IREC; CSIC-UCLM), Ciudad Real, Spain; ^4^ Department of Animal Health, Faculty of Veterinary Medicine, Complutense University of Madrid, Madrid, Spain, ucm.es

**Keywords:** African swine fever virus, high virulence isolate, histopathology, immunohistochemistry, low virulence isolate, vaccine, virus distribution, wild boar

## Abstract

Although several biosecurity and control measures are currently in place to mitigate the African swine fever (ASF) epidemic, vaccination is being explored as a potential long‐term strategy. However, standardized guidelines for evaluating the safety and efficacy of ASF vaccines are not yet fully established. Understanding infection dynamics in wild boar is crucial, as they play a key role in the spread and persistence of the virus. This work aims to provide comprehensive information on viral distribution through immunohistochemical analysis (p72) with histopathologic assessment in wild boar. The study design comprises animals: (i) intramuscular infected with the high‐virulence genotype II isolate Arm07 (highly virulent isolate [HVI]; *n* = 6); (ii) orally vaccinated with the low virulence isolate Lv17/WB/Rie1‐*Δ*CD (low virulent isolate [LVI]; *n* = 6); and (iii) orally vaccinated with Lv17/WB/Rie1‐*Δ*CD, either with a single dose (LVI‐HVI1; *n* = 6) or repeated doses (LVI‐HVI2; *n* = 6), followed by intramuscular challenge with Arm07. Clinical monitoring, viral load quantification in blood and tissues via real‐time quantitative PCR, and virus viability in tissue cultures using peripheral blood mononuclear cells were performed. HVI animals had hemorrhagic and inflammatory lesions, along with generalized lymphoid depletion, correlated with widespread viral dissemination. LVI animals rarely showed mild lymphoid depletion of the lymph nodes; minimal immunostaining was observed in macrophages of the tonsils and lymph nodes, typically restricted to the oral entry point. A few LVI–HVI1 cases had infected resident sinus macrophages related to necrotic lesions at tonsils and lymph nodes, preventing the virus from disseminating to vital organs. No viral immunostaining or associated histopathologic lesions were observed in LVI–HVI2 animals, indicating that revaccination enhances safety against virulent challenges. Observed changes following vaccination do not reflect chronic infection but rather a transient one, followed by lymphoid system recovery. Immunohistochemical and histological evaluation has proven valuable in advancing our understanding of ASF pathogenesis in wild boar, contributing to improved vaccination safety and disease management strategies.

## 1. Introduction

African swine fever (ASF) is a highly contagious and fatal hemorrhagic disease affecting domestic pigs and wild boar (*Sus scrofa*), included in the World Organization for Animal Health (WOAH) list of notifiable diseases [1]. The infection, caused by the *Asfarviridae* family virus (ASFV) [2], leads to severe economic losses and disruption of global pork trade [3, 4]. Although 24 ASFV genotypes have been identified [5–7], genotype II is of particular concern, as it was responsible for the re‐emergence of ASF in the Caucasus region and its subsequent spread across Europe, Asia, Africa, and the Americas [8, 9].

Wild boar play a crucial role in ASF maintenance and transmission, especially in Europe [10, 11]. Despite being more susceptible to ASFV infection than domestic pigs [12–14], they act as an important reservoir, facilitating viral persistence in the environment. Their natural wide‐ranging movements and interactions with domestic pigs can result in spillover events, complicating control efforts and increasing transmission risks [15, 16].

Vaccination is regarded as one of the most promising complementary strategies for the prevention and control of ASF. However, despite extensive research, the development of effective vaccines remains limited due to the virus’s genetic complexity and strain variability [8, 17, 18]. To date, two live attenuated vaccines have been approved for domestic use in Vietnam [19, 20], yet their long‐term safety, side effects, and cross‐protective efficacy remain uncertain.

A major challenge in ASF vaccine development is the absence of harmonized international guidelines for evaluating the quality, safety, and efficacy of candidate vaccines [21]. The assessment of live attenuated vaccines primarily relies on clinical data, viral replication, and monitoring of virus shedding [22–24]. Although real‐time PCR remains the gold standard for detecting ASFV genomic DNA [8], it does not provide insights into viral activity or tissue‐level effects. Therefore, complementary diagnostic approaches such as histopathology and immunohistochemistry (IHC) are essential to evaluate the pathogenesis and dynamics of infected macrophages, which are key players in ASFV infection [25, 26]. Moreover, IHC provides valuable information on the spatial distribution of viral antigen and its correlation with ASF‐associated lesions [27, 28]. Integrating these tools within a multifaceted approach is particularly important for evaluating vaccine safety [29], as it provides critical data on protective immunity, which is essential to prevent adverse reactions and ensure long‐term protection against evolving viral threats [19].

Few studies have described antigen viral detection in wild boar using IHC [30–33]; however, no attenuated ASFV isolate has been evaluated in this way. This study assessed, for the first time, the immunohistochemical localization of ASFV in wild boar orally vaccinated with the double‐deletion mutant Lv17/WB/Rie1‐*Δ*CD (single or repeated doses) [23], followed by intramuscular challenge with the highly virulent Arm07 strain. Combined with histopathological findings, these data allow evaluation of both the safety and protective capacity of the vaccine under different dosing protocols. To support this aim, histopathology and viral antigen distribution were analyzed across all organs in wild boar infected with Arm07 at 7 or 10 days postinfection (dpi), and in wild boar exposed only to Lv17/WB/Rie1‐*Δ*CD at 30 days postvaccination (dpv) to evaluate potential pathogenic effects of the attenuated virus.

In this study, clinical and macroscopic assessments, measurement of viremia, viral loads in target organs, and virus isolation from PBMC cultures were performed as complementary analyses. Together, these diagnostic tools provide comprehensive insights into ASF disease progression and are critical for evaluating the safety and efficacy of vaccine candidates, as well as for optimizing vaccination protocols. Integrated analyses also enhance our understanding of vaccine‐induced protection and tissue‐level effects, supporting evidence‐based strategies for ASF management.

## 2. Material and Methods

### 2.1. Animals and ASFV Isolates

The animals used in this study were sourced from two separate experiments. All experiments were performed under biosafety Level 3 conditions at the VISAVET Centre at the Complutense University of Madrid, following European, national, and regional regulations and approved by the Ethics Committee of the Community of Madrid (references PROEX 124/18, 159/19, and 113.3/21). Twenty‐four wild boar piglets, aged 3–4 months and weighing 10–15 kg, were obtained from a commercial wild boar farm, which tested negative for the following main porcine pathogens in the region: *Mycobacterium bovis*, *Mycoplasma hyopneumoniae*, suid herpesvirus 1, and porcine circovirus type 2. Details of the virus isolates and antibody detection against ASFV have been previously described in [34, 35]. The specific details of each animal are shown in Supporting Information 1: Table S1.

The highly virulent hemadsorbing genotype II ASFV Armenia07 (Arm07) isolate was propagated in PBMCs, and the viral titer was defined as the amount of virus causing hemadsorption (HAd) in 50% of infected cultures (HAD50/mL). The isolate Lv17/WB/Rie1‐*Δ*CD isolate was used for vaccination, generated according to the previous method described in Gallardo et al. [23], and the viral titer was determined as the amount of the virus causing a cytopathic effect using immunoperoxidase staining in 50% of infected cultures (TCID50/mL).

### 2.2. Experimental Design

**Figure 1:**
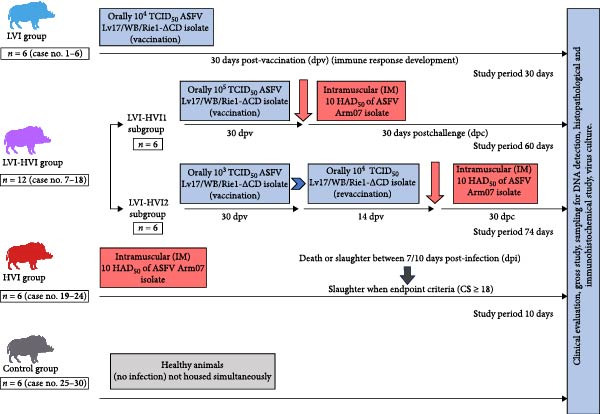
Scheme of the study design including the four groups (low virulent isolate [LVI], low virulent–high virulent isolate (LVI–HVI), high virulent isolate [HVI] and control group), from prime inoculation to the end of the experiment. All groups included in the experimental design underwent clinical evaluation, gross study, sampling for DNA detection, histopathological and immunohistochemical study, and virus culture. ASF, African swine fever; CS, clinical score; HAD50, median hemadsorption units; TCID50, median tissue culture infective dose.

Four different groups were included in this study: the low virulent isolate (LVI) group, the LVI and high virulent isolate (HVI; LVI–HVI) group, the HVI group, and the negative control group. The LVI group consisted of six animals orally vaccinated with 10^4^ TCID50 of the low virulent Lv17/WB/Rie1‐*Δ*CD isolate and sacrificed 30 days postvaccination (study period: 30 days). The LVI–HVI group included 12 animals orally vaccinated with the Lv17/WB/Rie1‐*Δ*CD isolate and subsequently challenged intramuscularly with 10 HAD50 of the highly virulent ASFV Arm07 isolate. This group was subdivided into two subgroups: the LVI–HVI1, composed of six animals vaccinated once with 10^5^ TCID50, in which the challenge was performed 30 days postvaccination, and animals were sacrificed 30 days after challenge (study period: 60 days); and the LVI–HVI2, composed of six animals initially vaccinated with 10^3^ TCID50 and revaccinated 30 days later with 10^4^ TCID50, in which the challenge was performed 14 days after revaccination and animals were sacrificed 30 days after challenge (study period: 74 days). The HVI group consisted of six animals infected intramuscularly with 10 HAD50 of the highly virulent ASFV Arm07 isolate and sacrificed 7 or 10 dpi, depending on the study endpoint (study period: 10 days). Finally, the negative control group included six healthy archival animals that were not infected or inoculated with any ASFV isolate and were maintained in separate facilities, without any temporal or physical contact with infected or vaccinated animals. Specific details of the experimental design, including timing of vaccination, revaccination, challenge, and sample collection, are shown in Figure 1.

### 2.3. Clinical Evaluation

Animals were observed daily throughout the study to monitor their health status by a video camera (recording 24 h a day) and visits by a wildlife‐specialist veterinarian. The evolution of the ASFV infection was assessed using a quantitative clinical score (CS) in accordance with specific parameters for ASFV infection in wild boar, described by Cadenas‐Fernández et al. [36]. All clinical observations were daily recorded, except temperature, to minimize animal handling and stress. The humane endpoint for this study was predefined as animals with a CS > 18. Additionally, inclusion criteria involved animals manifesting severe clinical signs at Level 4, including fever, altered behavior, compromised body condition, and persistent respiratory and digestive symptoms for over two consecutive days. Furthermore, euthanasia was considered for animals enduring unacceptable conditions, as determined by veterinarian criteria, irrespective of whether they reached the predefined humane endpoint. Only the CS obtained on the last day (day of slaughter) was considered for this study.

### 2.4. *Real-Time Quantitative Polymerase Chain Reaction* (*qPCR*)

During the postmortem examination, blood samples and a set of 17 selected tissues were collected. Seven of these were lymph nodes (renal, mediastinal, retropharyngeal, mesenteric, gastrohepatic, inguinal, and submandibular), as well as palatine tonsils, lungs, heart, liver, spleen, kidneys, urinary bladder, intestine, bone marrow, and brain. DNA extraction from all these samples was carried out using the High Pure PCR Template Preparation Kit (Roche Diagnostics GmbH, Roche Applied Science, Mannheim, Germany) to validate the presence of the ASFV genome in these samples. In brief, 10% (w/v) clarified homogenized tissue suspensions or blood samples were prepared in phosphate‐buffered saline. The detection of the ASFV DNA in blood and tissue samples was performed using the Universal Probe Library (UPL) qPCR recommended by the WOAH and previously described by Fernández‐Pinero et al. [37]. Based on the DNA sequence of the VP72 coding genome region of ASFV (GenBank Accession Number: S89966), the primers were prepared at a concentration of 20 pmol/µL, with the forward primer sequence being 5^′^‐CCC‐AGG‐RGA‐TAA‐AAT‐GAC‐TG‐3^′^ (ASF‐VP72‐F) and the reverse primer sequence being 5^′^‐CAC‐TRG‐TTC‐CCT‐CCA‐CCG‐ATA‐3^′^ (ASF‐VP72‐R). According to the WOAH manual, the qPCR protocol was optimized using the LightCycler480 Probes Master kit (Roche Applied Science). Positive qPCR results were determined by identifying the cycle threshold (Ct) values at which reporter dye emission appeared above the background within 40 cycles. Only the blood Ct values obtained on the last day (day of slaughter) were considered for this study. Positive and negative controls were used in DNA extraction and qPCR.

### 2.5. Gross Evaluation of ASFV Lesions and Sampling

During the postmortem survey, macroscopic lesions were evaluated following a previously published protocol by Rodríguez‐Bertos [38]. For the current study, tissue samples were taken from the following organs and fixed in 10% neutral‐buffered formalin (Panreac AppliChem ITW Reagents): lungs (cranial, middle, caudal, and accessory lobes), spleen, liver, kidneys (cortex, medulla, and renal pelvis), lymph nodes (cortex and medulla), bone marrow (red portion), palatine tonsil, thymus, heart (epicardium, myocardium, endocardium, and atrioventricular valve), adrenal glands, urinary bladder, gallbladder, pancreas (head, body, and tail), stomach (cardia, fundus, corpus, and pyloric antrum), small intestine (duodenum, jejunum, ileum, and ileocecal valve), large intestine (cecum, colon, and rectum), central nervous system (CNS) (cerebrum and cerebellum), reproductive system (ovaries, uterine ducts, and uterine body), synovial membrane, and diaphragm.

### 2.6. Histopathologic Evaluation

After fixation, samples were trimmed, dehydrated (Citadel 2000 Tissue Processor, ThermoFisher Scientific, Waltham, MA), and embedded in paraffin (HistoStar Embedding Workstation, Thermo Fisher Scientific) following standard procedures. Each tissue sample block was sectioned (FinesseME + Microtome, Thermo Fisher Scientific) and stained with hematoxylin and eosin (H&E) (Gemini AS Automated Slide Stainer, Thermo Fisher Scientific). Histopathological lesions were evaluated and scored following a previously published protocol by Porras et al. [39]. Several lesions/parameters were assessed based on the organ/tissue affected, resulting in an evaluation of 80 lesions/parameters in each animal. These lesions were semiquantitatively scored as follows: normal (0), mild (1), moderate (2), and severe (3). With this method, each animal’s total histopathologic score (HS) was calculated as a sum of each parameter on a 0–240 grading scale.

### 2.7. Immunohistochemical Study

Deparaffinization and antigenic retrieval were performed in the paraffin sections placed on positively charged glass slides. These steps were carried out using the Epredia PT module deparaffin and heat‐induced epitope retrieval (HIER). Endogenous peroxidase was blocked by immersing the samples in a 3% hydrogen peroxide in methanol solution (Panreac Química S.L.U.) for 15 min. Then, the samples were incubated with 2.5% Normal Horse Serum (ImmPRESS VR Horse AntiMouse IGG Polymer Kit, Vector Laboratories) for blocking (RTU) for 1 h. Slides were then incubated overnight at 4 °C with the ASFV VP72‐18BG3 primary mouse monoclonal antibody (1:100 dilution; Gold Standard Diagnostics). Positive and negative controls were included in each batch of slides. Specific primary antibody was replaced by tris‐buffered saline (PBS) and by a subtype‐matching IgG (Vector Laboratories) to serve as two primary antibody‐omit negative technique controls. A liver from a wild boar infected with the Arm07 ASFV isolate was used as a positive control. After overnight incubation, the secondary antibody was added (ImmPRESS VR Horse AntiMouse/AntiRabbit IGG Polymer Kit, Peroxidase; Vector Laboratories) and incubated for 1 h. Peroxidase was used for the revealing process (ImmPACT NovaRED Substrate Kit Peroxidase). Afterward, the samples were counter‐stained with hematoxylin (Gemini AS Automated Slide Stainer, Thermo Fisher Scientific).

To detect the presence of ASFV antigen, intense granular and diffuse cytoplasmic labeling were evaluated in combination and considered a positive immunoreaction. Light brown, diffuse, nongranular cytoplasmic labeling without adjacent lesions is considered a potentially nonspecific immunoreaction. A semiquantitative assessment of different specific cells (macrophages/monocytes, hepatocytes, epithelial cells, endothelial cells, mesangial cells) immunolabeled for ASF viral antigen (protein VP72‐18BG3) was performed and evaluated as follows: immunolabeled cells were counted in five adjacent, nonoverlapping fields under a high‐power field (HPF) magnification (400x). A score from 0 to 3 was assigned to each sample: (0) no presence of immunolabeled cells; (1) 1–10 immunolabeled cells; (2) > 10–50 immunolabeled cells; and (3) > 50 immunolabeled cells. With this method, each animal’s total immunohistochemical score (IHCS) was calculated as a sum of each parameter on a 0–75 grading scale.

### 2.8. Virus Culture

Virus isolation was performed on tissue samples to assess the presence of the infectious virus. Initially, a homogenate was prepared from the tissue samples by grinding them in a suitable buffer, ensuring the tissue was adequately disrupted for further analysis. The homogenate was first evaluated using qPCR to detect the viral genome and confirm the presence of the virus. Following this, the homogenate was subjected to an HAd assay using PBMCs, as outlined in the WOAH Terrestrial Manual [40]. The assay was carried out in 96‐well plates, with each sample tested in eight replicates to ensure reliable and reproducible results. The plates were monitored daily for HAd over a 6‐day period, and the presence of HAd or cytopathic effect was recorded as a sign of successful infection. To ensure the virus was viable and capable of replication, the samples underwent three blind passages. Each passage was followed by parallel qPCR testing to monitor viral load and confirm virus replication.

### 2.9. Statistical Analysis

Animal HS and IHCS, along with the Ct values for the blood and all the tissues evaluated, were compared between groups. Analyses were performed using a Kruskal–Wallis (K‐W) test followed by a Mann–Whitney (M‐W) test for pairwise multiple comparisons. Spearman’s rank correlation coefficient (*ρ*) was used to assess the relationships between HS, IHCS, and Ct values in blood and tissues within each animal group. All statistical analyses were performed in IBM SPSS Statistics for Windows, version 287.0. 1. 1 [15] (IBM Corp, Armonk, N.Y, United States). Statistically significant differences in all tests were considered when the *p*‐value was <0.05. Graphics were performed in R Statistical Software [41].

## 3. Results

### 3.1. Viral Loads in Blood and Tissue Samples

At 30 dpv, the LVI group showed low viral loads in blood (Ct = 35.8; 95% CI: 32.7–38.6) and tissues (Ct = 38.5; 95% CI: 38.1–39), mainly in lymph nodes (submandibular, retropharyngeal, inguinal, mediastinal), palatine tonsils, lungs, spleen, kidneys, and urinary bladder. The negative control group showed no viral detection in blood or tissues. The LVI–HVI group showed subgroup‐dependent differences. At 30 dpc, the LVI–HVI1 subgroup had low to moderate viral loads in blood (Ct = 28.9; 95% CI: 24.3–33.4) and tissues (Ct = 35.3; 95% CI: 34.4–36.1, highlighting the viral dissemination in almost all tissues in cases nos. 7, 9, and 10. In addition, case no. 8 displayed exceptionally high viral loads in both blood (Ct = 17.4) and all tissues (Ct = 20.7; 95% CI: 18.6–23.9). In the LVI–HVI2 subgroup, viral loads were minimal or undetectable in blood (Ct ≥ 40) and low in tissues (Ct = 38.8; 95% CI: 36.3–40), primarily in lymph nodes, palatine tonsils, lungs, liver, and spleen; the exception was case no. 14, which showed low viral loads in blood (Ct = 32.6) and most tissues (Ct = 35.7; 95% CI: 34.3–37.1). At 7–10 dpi, the HVI group exhibited high viral loads in blood (mean Ct = 18.1; 95% CI: 15.3–21) and all tissues (Ct = 21.3; 95% CI: 20.6–22).

**Figure 2:**
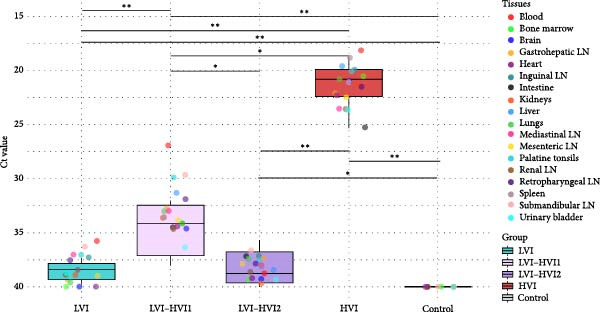
African swine fever virus Ct (cycle threshold) values (tissues) obtained in each group (low virulent isolate [LVI] group = clear blue color; low virulent‐high virulent isolate [LVI–HVI1] subgroup = clear purple color, low virulent‐high virulent isolate [LVI–HVI2] subgroup = dark purple color; high virulent isolate [HVI] group = red color; control group = gray color). Boxes indicate the interquartile range, middle highlighted bars inside boxes indicate median values, and the top and bottom of the box indicate the 75th and 25th percentiles. Whiskers denote the 97.5th and 2.5th percentiles. LN, lymph node.  ^∗∗∗^ *p*  < 0.001;  ^∗∗^ *p*  < 0.01;  ^∗^ *p*  < 0.05.

Statistically significant differences in viral loads were observed between groups (K‐W test, *p* < 0.01). The *U* M‐W analysis revealed significant differences between the HVI group and LVI and negative control groups (M‐W test: *p* < 0.01), between the HVI group and LVI–HVI1 subgroup (M‐W test: *p* < 0.05), and between HVI and LVI–HVI2 subgroup (M‐W test: *p* < 0.01). Significant differences were observed between both LVI‐HVI subgroups (M‐W test: *p* < 0.05), and between LVI and LVI–HVI1 groups (M‐W test: *p* < 0.01), but no differences were found between LVI and LVI–HVI2 groups. Mean Ct values in each group (blood and tissues) and detailed statistically significant differences are shown in Figure 2.

### 3.2. Clinical and Gross Evaluations

The LVI animals presented a 0% mortality rate and presented no relevant clinical signs other than mild transient fever. Necropsy findings showed mild and nonespecific gross lesions, mainly mild hyperemic splenomegaly with apparent white pulp hypertrophy (6/6), slight hepatomegaly with congestion (3/6), and occasional and minimal subpleural multifocal petechial hemorrhages (3/6) (Figure 3A).

Figure 3Necropsy findings of wild boar infected with low and high African swine fever virus isolates. Thoracic and abdominal cavities from (A) low virulent isolate (LVI) group, (B) low virulent–high virulent isolate (LVI–HVI1) subgroup, (C) low virulent–high virulent isolate (LVI–HVI2) subgroup, and (D) high virulent isolate (HVI) group. (A–C) Hyperemia and enlargement of the spleen (arrowhead); subpleural petechias (arrow), and (B) multifocal red consolidated areas (large arrowhead). (D) Moderate hydrothorax with interstitial and alveolar pulmonary edema, congestion, and patchy multifocal hemorrhages (arrow); multifocal hemorrhages in the diaphragm (large arrowhead), and hyperemia and marked enlargement of the spleen (arrowhead).

**Figure:**
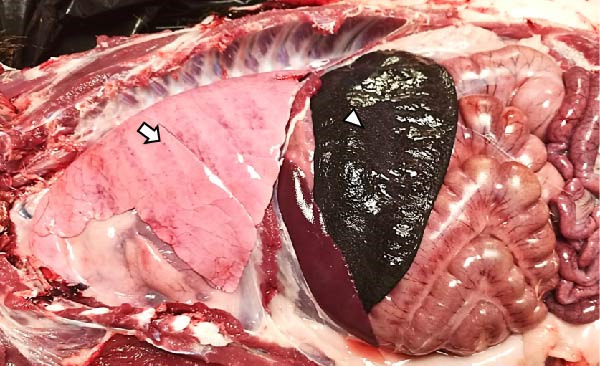
(A)

**Figure:**
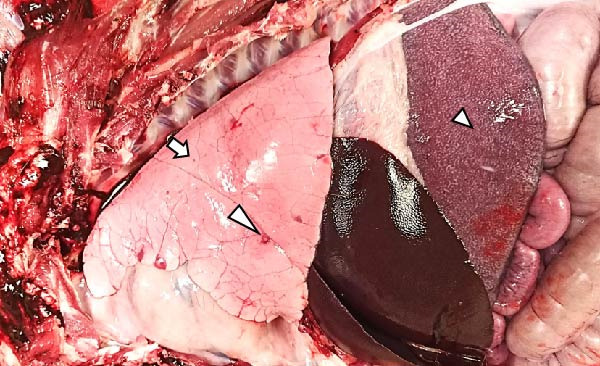
(B)

**Figure:**
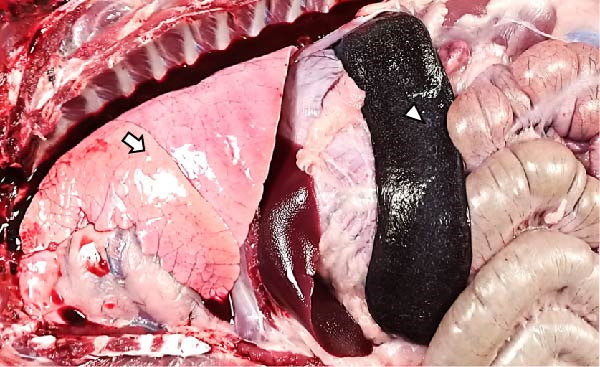
(C)

**Figure:**
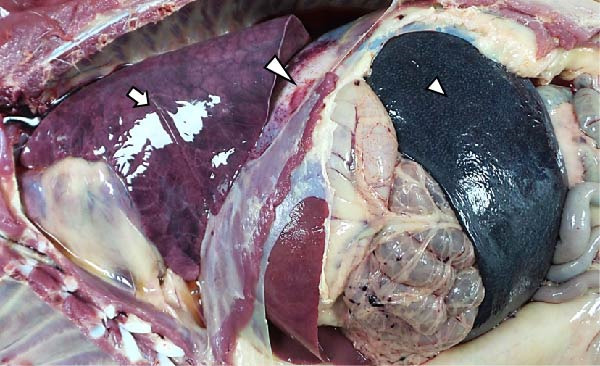
(D)

The LVI–HVI group showed an overall mortality rate of 16.7% (two out of 12). One nonimmunized animal from the LVI–HVI1 subgroup (case no. 8) developed acute ASF disease after challenge, while one LVI–HVI2 animal (case no. 17) was euthanized due to severe stress‐related injuries, presenting lesions compatible with traumatic aggressions. The remaining animals displayed only transient fever during viraemia, more pronounced in the LVI–HVI1 subgroup. Animals from both LVI–HVI subgroups exhibited predominantly mild and nonspecific gross lesions, although those from LVI–HVI1 were generally more frequent and pronounced. Common findings included mild ascites (2/5 per subgroup), slight splenomegaly (2/5 in each subgroup), hepatomegaly (1/5 in both), scattered subpleural petechial hemorrhages (3/5 in both), petechial hemorrhages in the epicardium (1/5 in both), and mild lymphadenomegaly of the gastrohepatic, renal, and mesenteric lymph nodes (1/5 in each) (Figure 3B,C). In addition, one animal from LVI–HVI2 showed mild edema of the gallbladder wall with serohemorrhagic content.

Animals in the HVI group exhibited 100% mortality and clinical signs consistent with acute ASF, including elevated body temperature, lethargy, walking difficulties, widespread erythema, mild ocular discharge, and digestive disturbances such as mucus in stools and occasional vomiting. Two IM‐infected animals were euthanized at 7 dpi according to humane endpoint criteria, while the remaining animals succumbed naturally between 7 and 10 dpi. Necropsy revealed moderate to severe ascites, hydrothorax, and hydropericardium, along with pulmonary edema, congestion, and multifocal subpleural hemorrhages. Marked splenomegaly, hepatomegaly, and generalized lymphadenomegaly were also observed (Figure 3D). Hemorrhages of varying severity were frequently present in lymph nodes and kidneys and occasionally affected other organs, including the thymus, heart, urinary bladder, adrenal glands, pancreas, and gastrointestinal mucosa. No relevant clinical signs or gross lesions were observed in the negative control group, aside from nonspecific splenomegaly and/or lymphadenomegaly.

### 3.3. Histopathological Evaluation

Animals in the LVI group exhibited predominantly nonspecific lesions, with minimal vascular changes, including congestion and mild erythrocyte extravasation in multiple lymph nodes (retropharyngeal, gastrohepatic, renal, and mesenteric) as well as in thymus, lungs, liver, spleen, stomach, intestines, and brain. Mild mononuclear inflammatory infiltrates were observed in the tonsils, lungs, liver, kidneys, urinary bladder, and gastrointestinal tract. Some animals showed minimal lymphoid depletion and lymphocyte apoptosis, particularly in germinal centers of lymph nodes, tonsils, and spleen, often accompanied by moderate mitotic activity. Thymic cortical atrophy was minimal, with limited tingible body macrophages (TBMs), while bone marrow exhibited mild granulocytic hyperplasia and slight reduction in megakaryocytes (Figure 4). Spearman’s correlation analysis revealed a significant negative association between histopathological score (HS) and blood Ct values (*ρ* = −0.882, *p* < 0.05).

Figure 4Histopathologic findings in lymphoid tissues of wild boars exposed to the ASF low virulent isolate (LVI group). (A) Palatine tonsils. The epithelium of the crypt contains a reduced number of pyknotic cells and karyorrhexis with mild infiltration of lymphocytes (arrowhead); the lumen of the crypt is filled with eosinophilic material mainly composed of epithelial cell debris, and mild number of degenerated lymphocytes and tingible body macrophages (arrow). Hematoxylin and eosin (H&E). (B, C) Submandibular and gastrohepatic lymph nodes. There is a mild to moderate hypocellularity of the lymphocytes that composed the lymphoid follicles; the mantle zone (arrowhead) is mildly depleted and difficult to discern with the germinal center. H&E. (D) Lymph node, germinal center. Multifocal lymphoid depletion with minimal number of cell debris (arrow) and pyknotic cells (large arrowhead), and mild to moderate number of mitotic cells (arrowhead). H&E.

**Figure:**
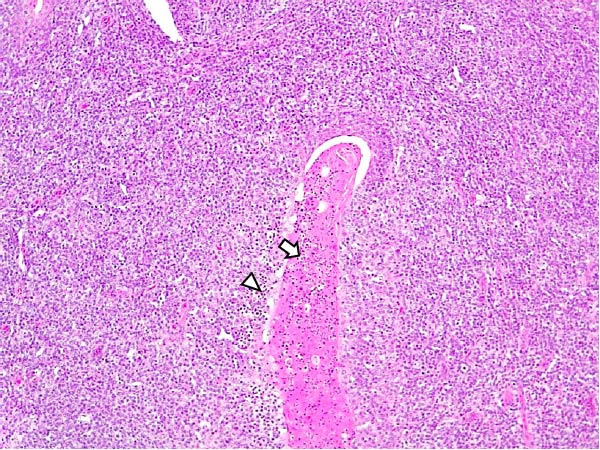
(A)

**Figure:**
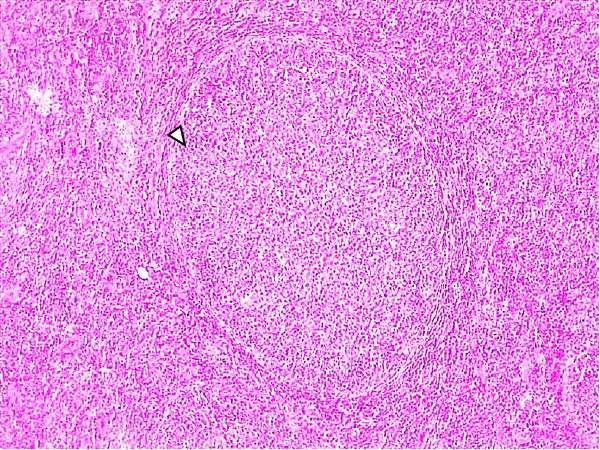
(B)

**Figure:**
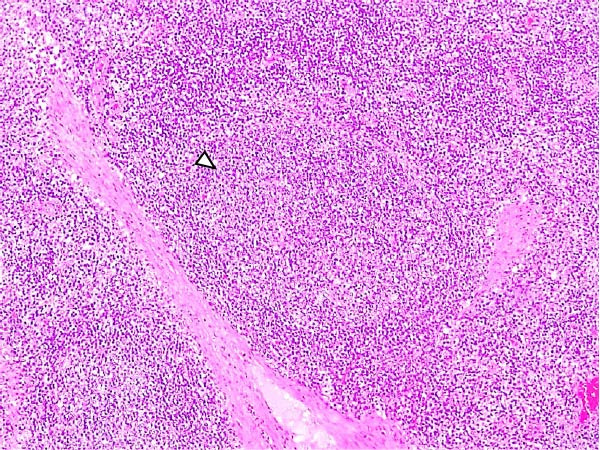
(C)

**Figure:**
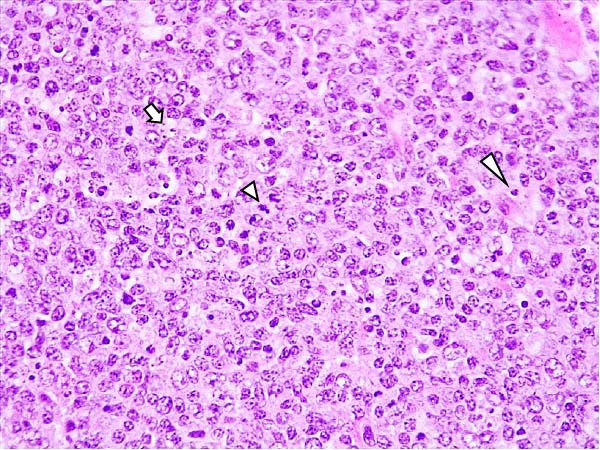
(D)

Lesions in the LVI–HVI subgroups were generally mild and nonspecific, primarily involving congestion and erythrocyte extravasation in lymph nodes (submandibular, retropharyngeal, gastrohepatic, mediastinal, renal, mesenteric, ileocecal), liver, and lung. Minimal inflammatory infiltrates were observed in the tonsils, lungs, liver, and gastrointestinal tract, while bone marrow exhibited slight myeloid hypocellularity and reduced megakaryocyte numbers. A significant difference in lymphoid depletion was observed between the two subgroups. Case nos. 7 and 10 (LVI–HVI1) displayed mild multifocal lymphoid necrosis, surrounded by a moderate number of TBM, adjacent to the trabecular or medullary sinuses, mainly at palatine tonsils, submandibular, mediastinal, ileocecal, gastrohepatic, and renal lymph nodes (Figure 5A,C,E). Spearman’s rank correlation analysis revealed a significant negative correlation between HS and tissue Ct values in the LVI–HVI1 subgroup (*ρ* = −0.829, *p* < 0.05), but also in the LVI–HVI2 subgroup (*ρ* = −0.943, *p* < 0.01). No necrotic lesions or lymphoid depletion were observed in the LVI–HVI2 subgroup (Figure 5B,D,F).

Figure 5Histopathologic findings in lymphoid tissues of wild boars exposed to the ASF low virulent isolate and challenged with the ASF high virulent isolate (LVI–HVI group). (A, C, E) LVI–HVI1 subgroup; (B, D, F) LVI–HVI2 subgroup. (A, B) Palatine tonsils. (A) The crypt loses its architecture. The epithelial cells disappear and are replaced by eosinophilic necrotic material filling the lumen (arrow). This material is composed of a moderate amount of cellular debris, degenerated lymphocytes, and tingible body macrophages (TBM). In the adjacent lymphoid tissue, surrounding the crypts, there is an intense infiltration of tingible body macrophages giving a starry‐sky appearance (arrowhead). Inset: TBM infiltration (arrowhead) surrounding the necrotic cells of the crypt. Hematoxylin and eosin (H&E). (B) The epithelium of the crypt has a reduced number of apoptotic cells (arrowhead), and the lumen of the crypt is filled with a moderate number of degenerated inflammatory cells (arrow). (C, D) Submandibular lymph nodes. (C) Areas of necrosis adjacent to the marginal zone (arrow) surrounded by an abundant number of TBM (arrowheads), have also been infiltrated in the germinal center. There is a mild to moderate hypocellularity of the lymphocytes that composed the germinal center. Inset: degenerated TBM replacing the cell composition of the lymphoid follicle (arrowhead). H&E. (D) Primary lymphoid follicle, observing a well‐defined structure, without lymphoid cell reduction (arrowhead). H&E. (E, F) Gastrohepatic lymph node. (E) Areas of necrosis adjacent to the trabecular sinus (arrow), surrounded by an abundant number of TBM (arrowhead). Inset: degenerated inflammatory cells and lymphoid cell debris surrounding the necrotic area (arrowhead). H&E. (F) The germinal center is reactive, observing a well‐defined mantle zone (arrowhead). H&E.

**Figure:**
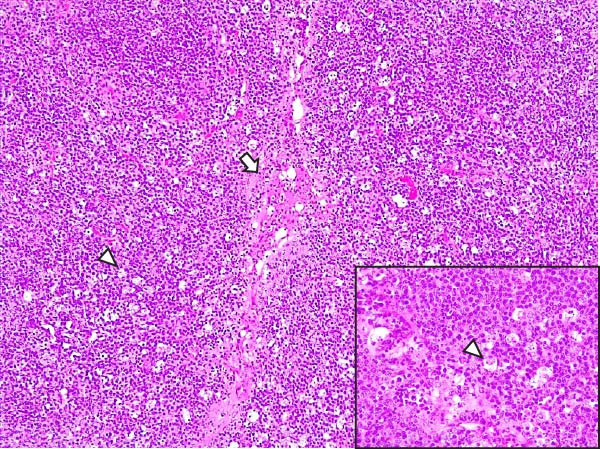
(A)

**Figure:**
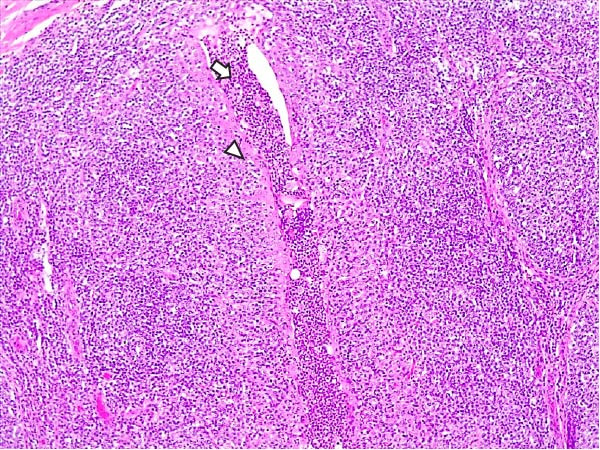
(B)

**Figure:**
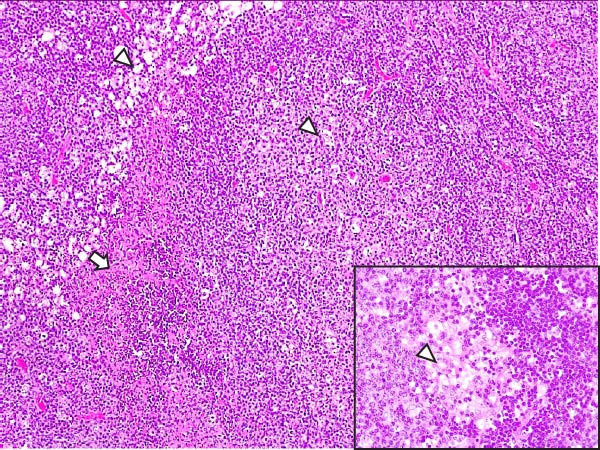
(C)

**Figure:**
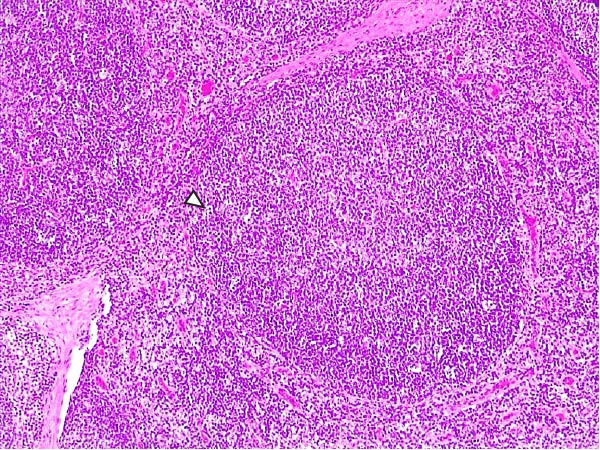
(D)

**Figure:**
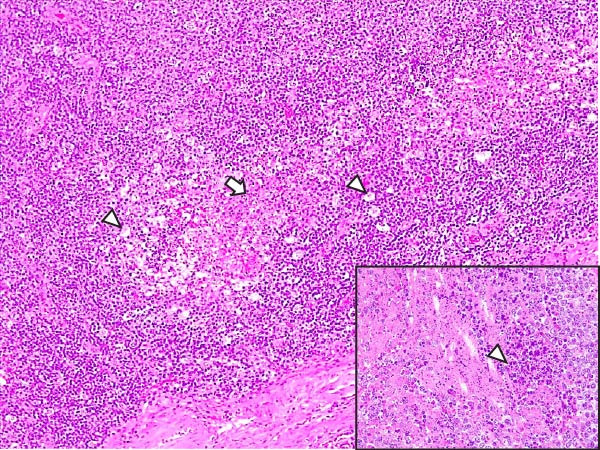
(E)

**Figure:**
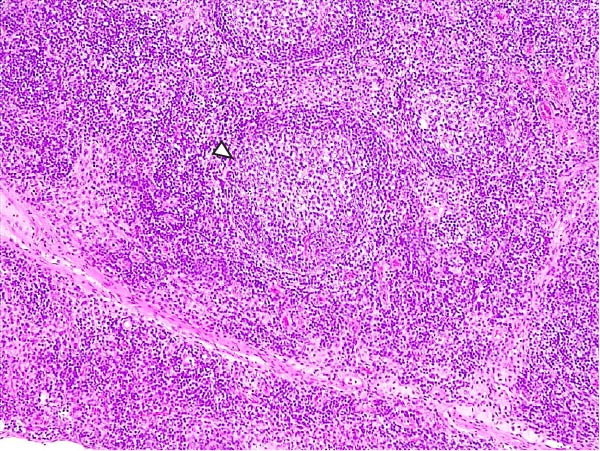
(F)

Two animals in the LVI–HVI group showed more pronounced lesions. Case no. 8 (LVI–HVI1 subgroup) exhibited histological changes consistent with an early acute form of ASF, resembling those observed in the HVI group. In contrast, case no. 17 (LVI–HVI2 subgroup), which had suffered severe traumatic injuries, displayed lesions only partially compatible with ASF. These included moderate lymphoid atrophy and multifocal lymphocytolysis in the spleen, lymph nodes, tonsils, and GALT, together with mild to moderate hemorrhagic and congestive changes in several organs (lungs, liver, kidneys, adrenal gland, and thymus).

Infrequent additional lesions, including granulomas, Splendore‐Hoeppli bodies, parasitic pneumonia, or intestinal microabscesses, were occasionally present and could bias the ASF‐specific HS.

Animals in the HVI group exhibited the typical acute form of ASF, characterized by extensive lymphoid depletion and lymphocytolysis in the tonsils, thymus, spleen, bone marrow, lymph nodes, and mucosa‐associated lymphoid tissue. Intense congestion and multifocal hemorrhages were observed in multiple organs, particularly in the gastrohepatic and renal lymph nodes, spleen, lungs, liver, kidneys, adrenal glands, urinary bladder, and brain (notably in the choroid plexus). Mononuclear inflammatory infiltrates were also evident in the lungs, liver, gastrointestinal tract, and brain, together with necrotizing and suppurative tonsillitis. As infection progressed (up to 10 dpi), lesion severity increased, with target organs (lungs, spleen, liver, kidneys, urinary bladder, gallbladder, adrenal glands, and pancreas) showing more extensive hemorrhagic and inflammatory changes. At this stage, pronounced lymphoid depletion and lymphocytolysis were particularly evident in the palatine tonsils, spleen, and lymph nodes. Spearman’s rank correlation analysis revealed a significant negative correlation between HS and blood Ct values (*ρ* = −0.829, *p*  < 0.05) in the HVI group. Statistically significant differences in HS values were observed between groups (K‐W test: *p* < 0.001). The *U* M‐W analysis revealed significant differences between the HVI group and LVI and negative control groups (M‐W test: *p* < 0.01) and between the HVI group and LVI–HVI groups (M‐W test: *p* < 0.01). No significant differences were observed between the LVI–HVI subgroups, nor between the LVI and LVI–HVI groups. HS values in each group and detailed statistically significant differences are shown in Figure 6A. See Table 1 for a comparative summary of key histopathological findings.

Figure 6(A) Mean histopathologic score (HS) and (B) immunohistochemical score (IHCS), obtained according to the group (low virulent isolate (LVI) group = clear blue color; low virulent–high virulent isolate (LVI–HVI1) subgrou = clear purple color, low virulent‐high virulent isolate (LVI‐HVI2) subgroup = dark purple color; high virulent isolate (HVI) group = red color; control group = gray color). Boxes indicate the interquartile range, middle highlighted bars inside boxes indicate median values, and the top and bottom of the box indicate the 75th and 25th percentiles. Whiskers denote 97.5th and 2.5th percentiles. Single points represent each individual. The HS and IHCS of each animal and tissue were obtained by summing the scores calculated through semiquantitative evaluation (0–3) of the different tissues.  ^∗∗∗^ *p*  < 0.001;  ^∗∗^ *p*  < 0.01;  ^∗^ *p*  < 0.05.

**Figure:**
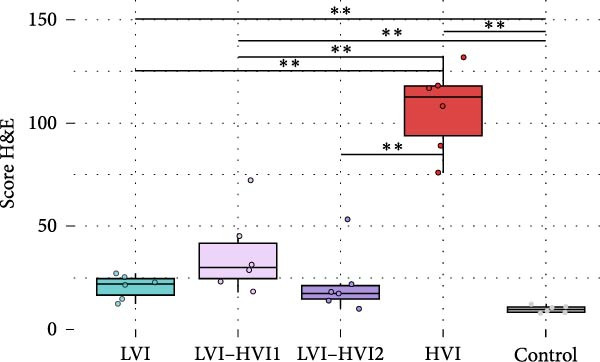
(A)

**Figure:**
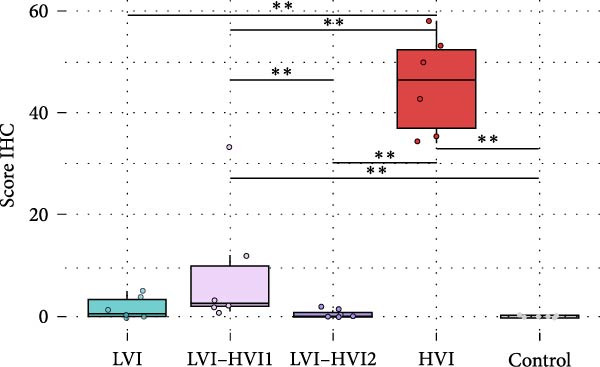
(B)

**Table 1:** Overview of key histopathological lesions and main ASFV p72 antigen distribution by IHC in each wild boar group.

Group	Main histopathological findings	Main ASFV p72 detection (IHC)
LVI	Mild mononuclear infiltration, congestion, and erythrocyte extravasation in lymph nodes, tonsils, thymus, spleen, bone marrow, lungs, liver, kidneys, gastrointestinal tract, and brain. Minimal lymphocytolysis and lymphoid depletion of lymph nodes.	Minimally distributed, diffuse, and granular cytoplasmic immunoreaction in single macrophages and epithelial cells in crypts of tonsils, and macrophages in lymph nodes, kidney, urinary bladder, and synovial membrane.
LVI–HVI1	Mild multifocal crypt necrosis and lymphocytolysis in tonsils. Focal lymphocytolysis and TBM infiltration in lymph nodes in subcapsular, trabecular, or medullary sinuses (2/6; case nos. 7 and 10). Early acute ASF lesions with marked lymphoid depletion and multifocal lymphocytolysis in tonsils, spleen, lymph nodes, bone marrow, and GALT; hemorrhages in main lymph nodes; congestion in lung, liver, and kidney (1/6; case no. 8).	Mild to moderately distributed, diffuse, and granular cytoplasmic immunoreaction, with inclusion bodies (in:) macrophages of tonsils adjacent to lymphocytolysis, TBM and necrotic crypts; and in macrophages of lymph nodes, associated with lymphocytolysis and TBM (2/6; case nos. 7 and 10). Moderate to intensely distributed, diffuse and granular immunoreaction, with inclusion bodies, in macrophages of tonsils, lymph nodes, spleen, bone marrow, lungs, liver, and kidneys (1/6; case no. 8).
LVI–HVI2	Lymph nodes and tonsils with no significant lymphocyte apoptosis (5/6). Lesions related to injuries, extensive subcutaneous hematomas, subcutaneous abscess, mild pulmonary edema, mild hemorrhages hemorrhages and congestion in lymph nodes, lungs, liver, kidneys, adrenal gland, and thymus. Moderate lymphoid depletion with multifocal lymphocytolysis (1/6; case no. 17).	Minimally distributed, diffuse cytoplasmic immunoreaction, in single macrophages of tonsils and submandibular lymph nodes, not associated with lesions (2/6; case nos. 14 and 17).
HVI	Intense lymphoid depletion and lymphocytolysis in tonsils, lymph nodes, spleen, thymus, and bone marrow; multifocal hemorrhages in lungs, liver, kidneys, heart, adrenal glands, GI tract, and brain. Moderate to severe mononuclear infiltrates in lungs, liver, GI tract, and brain. Alveolar edema and hemorrhages. At 10 dpi, lymphoid depletion and extensive hemorrhages are more intense.	Moderately distributed, diffuse and granular immunoreaction, with inclusion bodies, in macrophages of tonsils, lymph nodes, spleen, bone marrow, lungs, liver, and kidneys (3/6, 7 dpi). Increased and widespread immunoreaction in the same organs, with mild to moderate appearance in heart, gallbladder, GI tract, adrenal gland, urinary bladder, synovial membrane, and brain (3/6, 10 dpi).
Control	No relevant lesions; only minimal nonspecific changes.	Nonspecific diffuse cytoplasmic immunoreaction in macrophages, lymphocytes, and plasma cells in several lymphoid tissues and GI mucosa; not associated with lesions.

Abbreviations: dpi, days postinfection; GI, gastrointestinal; TBM, tingible body macrophage.

### 3.4. Immunohistochemical ASFV p72 Detection

In the LVI group, mild brown granular and diffuse cytoplasmic antigen immunostaining was detected in macrophages, mainly in the palatine tonsils (3/6) (Figure 7A). Occasional labeling was also observed in the spleen (1/6) (Figure 7B), lymph nodes (1/6) (Figure 7C), kidneys (1/6) (Figure 7D), and urinary bladder (1/6) (Figure 7E), where a single labeled monocyte/macrophage was observed within a blood vessel lumen. Rarely, diffuse cytoplasmic immunolabeling was seen in the synovial membrane (1/6) (Figure 7F), associated with mild perivascular mononuclear inflammation. A significant negative correlation was found between IHCS and tissue Ct values (*ρ* = –0.926, *p*  < 0.01).

Figure 7Immunohistochemical p72 ASF virus detection in wild boars exposed to the ASF low virulent isolate (LVI group). (A) Palatine tonsils. Granular and diffuse immunostaining in the cytoplasm of an apoptotic cell inside the crypt lumen (arrowhead). Inset: granular immunostaining in the cytoplasm between epithelial cells of the crypt (arrow). (B) Spleen. Granular and diffuse immunostaining in the macrophage cytoplasm inside the red pulp (arrowhead). (C) Lymph node. Diffuse immunostaining in the macrophage cytoplasm in the medullary sinus (arrowhead). (D) Kidney. Granular and diffuse immunostaining in the cytoplasm of mononuclear inflammatory interstitial infiltrate (arrowhead). (E) Urinary bladder. Granular and diffuse immunostaining in the cytoplasm of a circulating monocyte/macrophage inside a capillary (arrowhead). (F) Synovial membrane. Granular and diffuse immunostaining in the cytoplasm of mononuclear inflammatory perivascular infiltrate (arrowhead).

**Figure:**
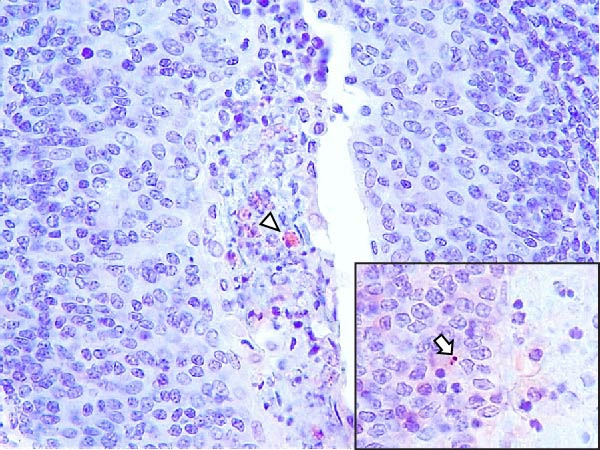
(A)

**Figure:**
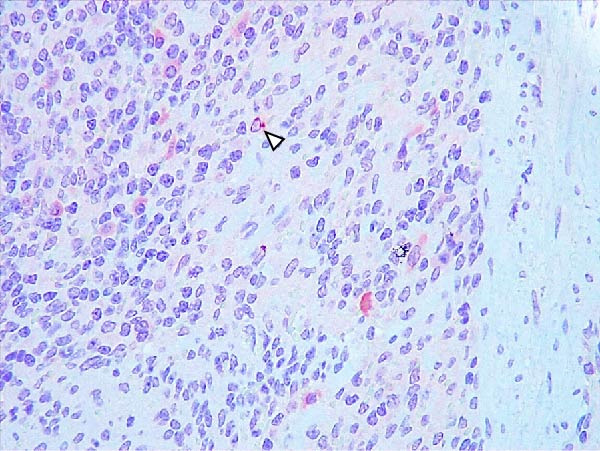
(B)

**Figure:**
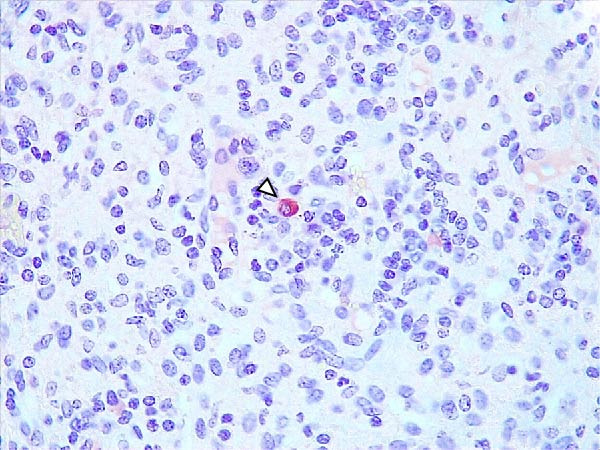
(C)

**Figure:**
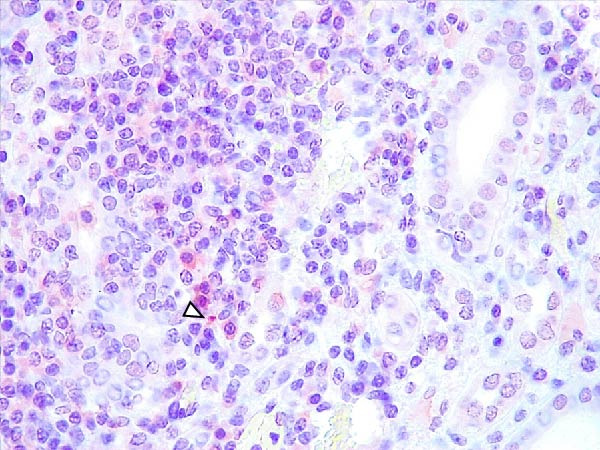
(D)

**Figure:**
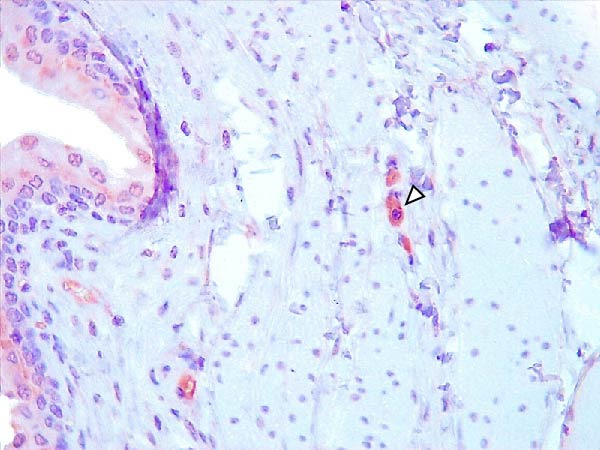
(E)

**Figure:**
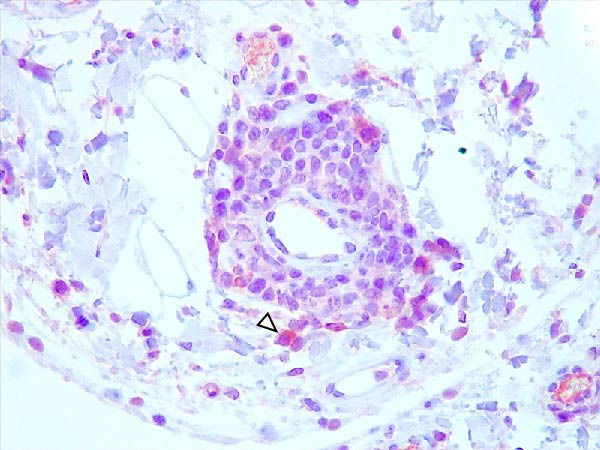
(F)

Case nos. 7 and 10 (2/6) from the LVI–HVI1 subgroup showed moderate granular cytoplasmic immunoreactivity in macrophages, with inclusion bodies, in the tonsils, associated with mild crypt necrosis and lymphocytic apoptosis. In these cases, similar immunolabeling was also observed in macrophages of several lymph nodes (submandibular, retropharyngeal, mediastinal, ileocecal, renal, and gastrohepatic), adjacent to subcapsular, trabecular, or medullary sinuses, and associated with focal lymphocytic apoptosis and TBM infiltration (Figure 8A,C,E). Exceptionally, case no. 8 (LVI–HVI1, nonimmunized) showed immunoreactivity typical of acute ASF. In contrast, immunostaining in the LVI–HVI2 subgroup was minimal, presenting as diffuse cytoplasmic labeling in isolated macrophages of the palatine tonsils and submandibular lymph nodes, without associated lesions (Figure 8B,D,F). Spearman’s correlation analysis revealed a significant positive association between IHCS and HS values in both subgroups (*ρ* = 0.986, *p*  < 0.01 for LVI–HVI1; *ρ* = 0.845, *p*  < 0.05 for LVI–HVI2) and a negative correlation with tissue Ct values in LVI–HVI1 (*ρ* = –0.899, *p*  < 0.05).

Figure 8Immunohistochemical p72 ASF virus detection in wild boars exposed to the ASF low virulent isolate and challenged with ASF high virulent isolate (LVI–HVI group). (A, C, E) LVI–HVI1 subgroup; (B, D, F) LVI–HVI2 subgroup. (A,B) Palatine tonsils. Intense granular and diffuse immunostaining in the cytoplasm of macrophages and tingible body macrophages (TBM) located inside and surrounding the necrotic area (arrowheads). (B) Granular and diffuse cytoplasmic immunostaining in a single macrophage inside the lymphoid tissue surrounding the crypt (arrowhead). (C, D) Submandibular lymph node. (C) Moderate granular and diffuse immunostaining in macrophages and TBM located surrounding the necrotic area (arrowheads). (D) Diffuse cytoplasmic immunostaining in a single macrophage inside the medullary sinus (arrowhead). (E, F) Gastrohepatic lymph node. (E) Intense granular and diffuse immunostaining in macrophages and TBM located inside and surrounding the necrotic area (arrowheads). (F) Granular and diffuse cytoplasmic immunostaining in a single macrophage inside the marginal zone (arrowhead). (B, D, F) The p72‐positive cells are not related to any lesion.

**Figure:**
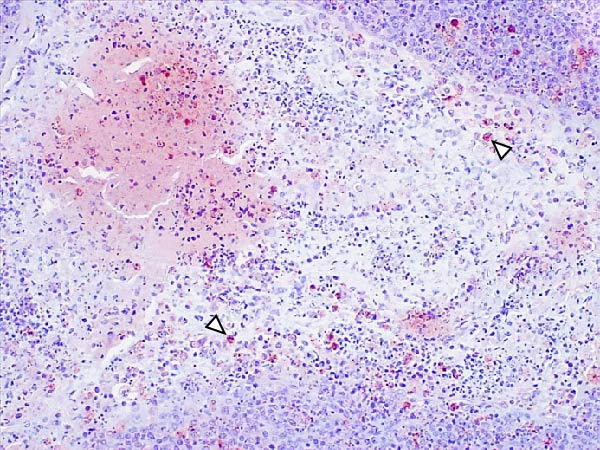
(A)

**Figure:**
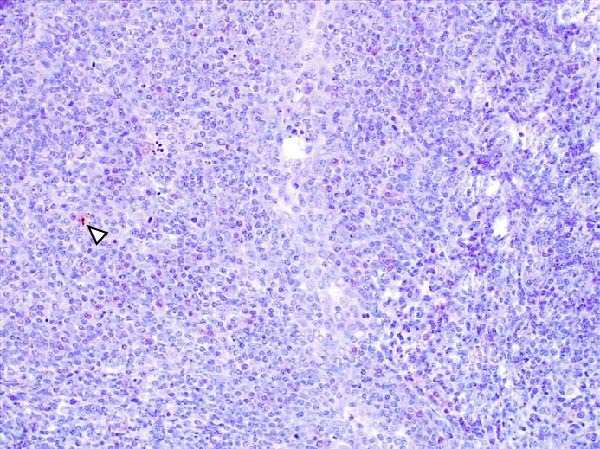
(B)

**Figure:**
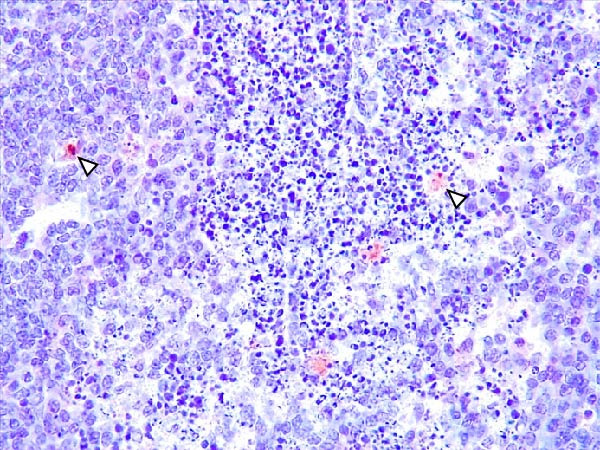
(C)

**Figure:**
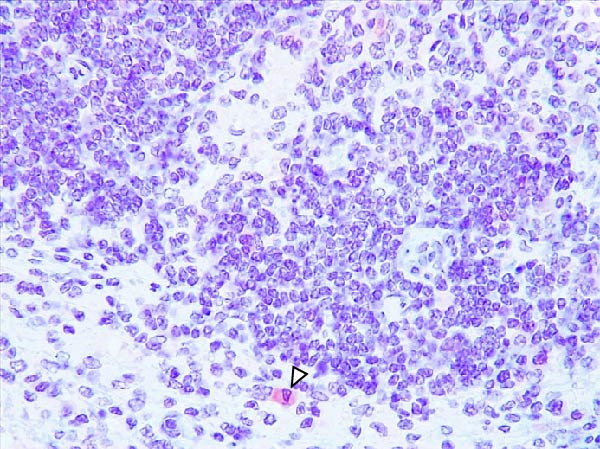
(D)

**Figure:**
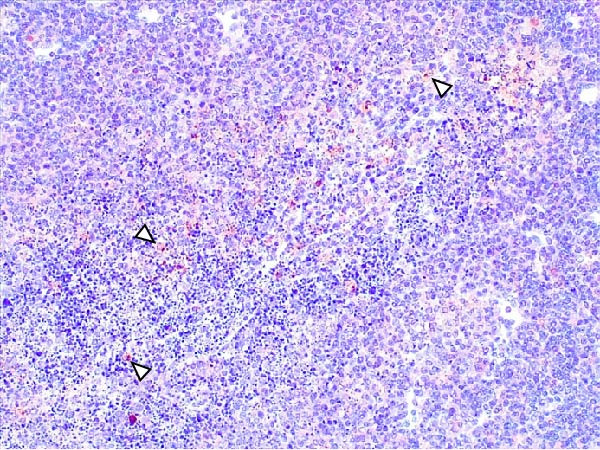
(E)

**Figure:**
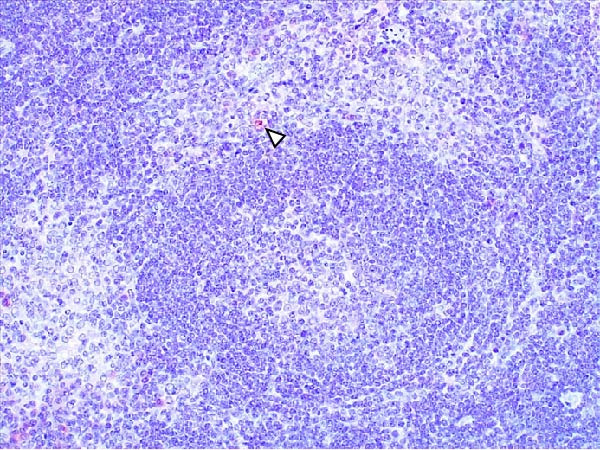
(F)

Animals in the HVI group exhibited strong and widespread granular cytoplasmic immunolabeling with frequent presence of inclusion bodies. Numerous p72‐positive macrophages infiltrated the crypt epithelium of the palatine tonsils and surrounding lymphoid tissue, with immunoreactive epithelial cells adjacent to necrotic or ulcerated crypts (Supporting Information 2: Figure S1A,B). Strong immunoreaction was also evident in interfollicular macrophages and occasionally within follicles. The thymus showed a moderate to severe number of p72‐positive macrophages, particularly at the corticomedullary junction and medulla (Supporting Information 2: Figure S1C,D). The bone marrow contained numerous labeled macrophages, monocytes, and endothelial cells, while megakaryocytes and granulocytes were rarely affected (Supporting Information 3: Figure S2A,B). In the spleen, abundant p72‐positive macrophages were found mainly in the red pulp and at the periphery of follicles (Supporting Information 3: Figure S2C,D). Antigen virus distribution in lymph nodes varied by location, with moderate immunolabeling in submandibular, retropharyngeal, and inguinal nodes, and intense, widespread immunoreaction in mediastinal, mesenteric, ileocecal, renal, and gastrohepatic nodes (Supporting Information 4: Figure S3 and Supporting Information 5: Figure S4). These infected macrophages were evident in medullary sinuses and interfollicular areas at 7 dpi (Figure 9A) and were more numerous at 10 dpi, particularly near areas of lymphocytolysis and within depleted follicles (Figure 9B).

Figure 9Immunohistochemical viral p72 detection in wild boars infected with ASF highly virulent isolate (HVI group) at 7 (A, C, E, G) and 10 (B, D, F, H) days postinfection, showing temporal differences in antigen distribution. (A, B) Gastrohepatic lymph node. (A) Mild granular and diffuse cytoplasmic immunostaining in macrophages located adjacent to medullary sinuses (arrowhead). Inset: ASF intracytoplasmic inclusion body (arrow). (B) Intense granular and diffuse cytoplasmic immunostaining in macrophages (arrowheads) located adjacent to depleted lymphoid follicles and surrounded by marked lymphocytolysis. Inset: ASF intracytoplasmic inclusion body in a macrophage (arrow). (C, D) Lungs. (C) Mild granular and diffuse cytoplasmic immunostaining in interstitial macrophages (arrowheads). Inset: ASF intracytoplasmic inclusion body in interstitial macrophage (arrow). (D) Intense granular and diffuse cytoplasmic immunostaining in interstitial macrophages (arrowheads). Inset: ASF positive intracytoplasmic inclusion body in macrophage inside the bronchus lumen (arrow). (E, F) Liver. (E) Minimal granular and diffuse cytoplasmic immunostaining in the centrilobular zone of the hepatic lobule (arrowhead). Inset: positive ASF intracytoplasmic inclusion body in Kupffer cell (arrow). (F) Intense granular and diffuse cytoplasmic immunostaining was multifocally spread in the entire hepatic lobule (arrowheads). Inset: ASF intracytoplasmic inclusion bodies in hepatocytes (arrow). (G, H) Kidneys. (G) Minimal granular and diffuse cytoplasmic immunostaining in the mesangial cell inside the glomerulus (arrowhead). (H) Intense granular and diffuse cytoplasmic immunostaining in the macrophages adjacent to interstitial mononuclear inflammatory infiltrate (arrowheads). Inset: ASF intracytoplasmic inclusion bodies in the mesangial cells/macrophages inside the glomerulus (arrow), and the interstitial macrophages (arrowhead).

**Figure:**
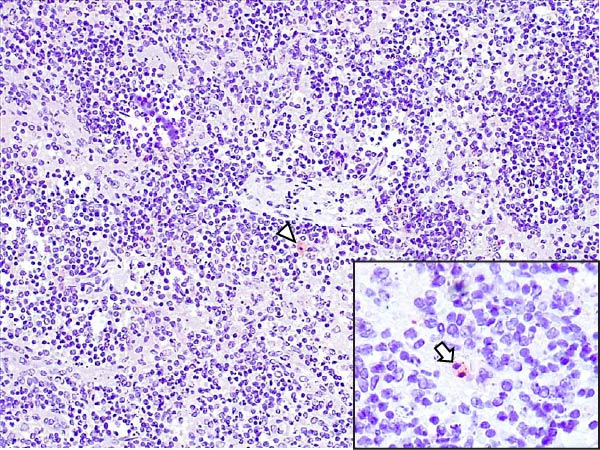
(A)

**Figure:**
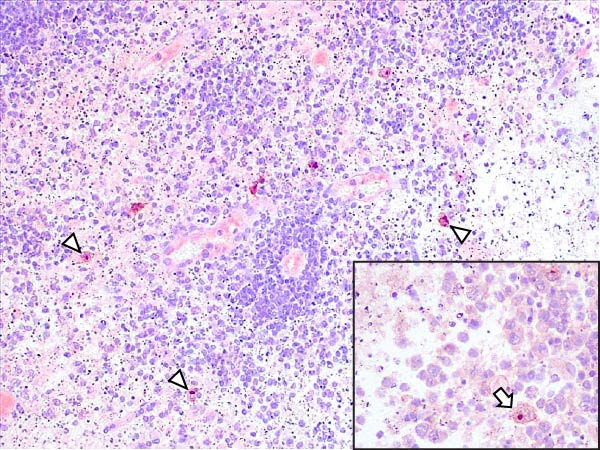
(B)

**Figure:**
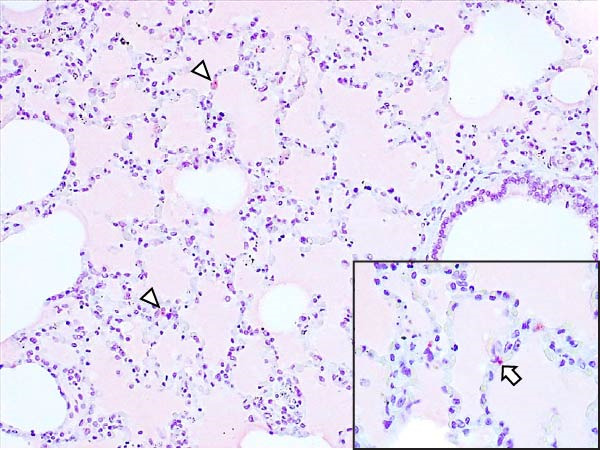
(C)

**Figure:**
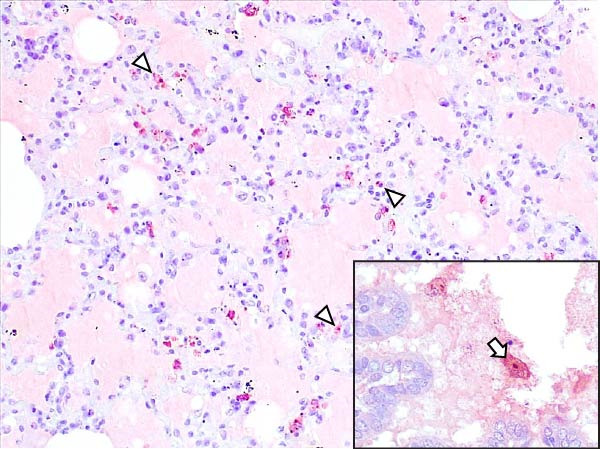
(D)

**Figure:**
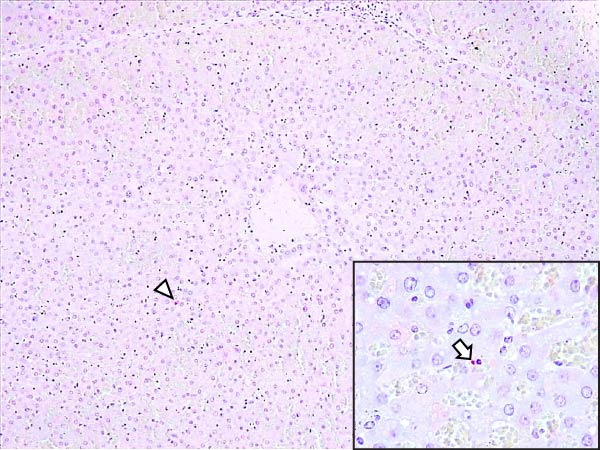
(E)

**Figure:**
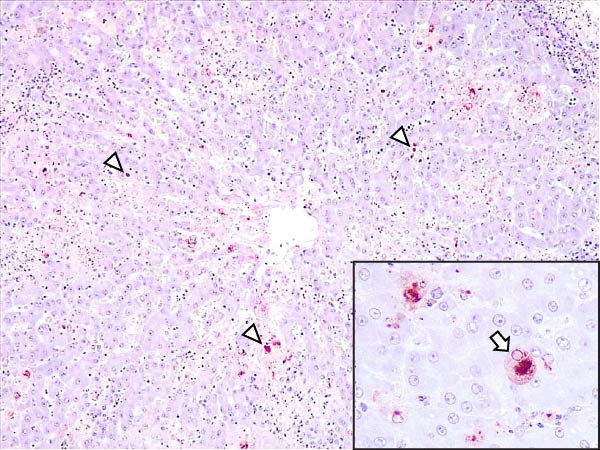
(F)

**Figure:**
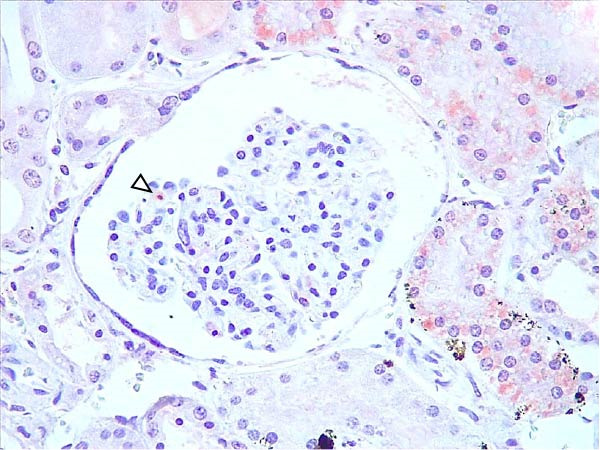
(G)

**Figure:**
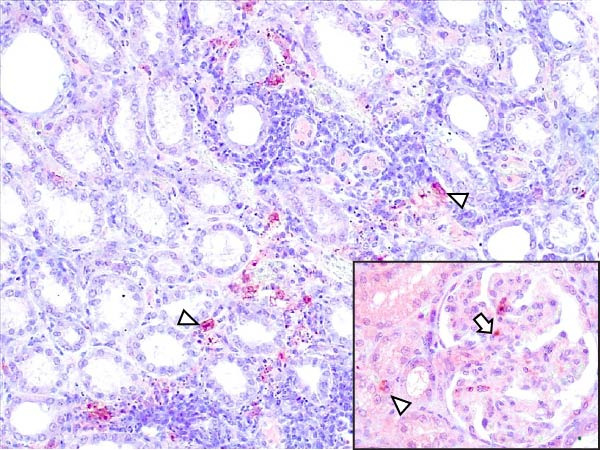
(H)

In the lungs, p72‐positive macrophages were scarce at 7 dpi but markedly increased at 10 dpi (Figure 9C,D). At later stages, p72 was frequently detected in pulmonary alveolar and intravascular macrophages, pneumocytes, and bronchial lumen macrophages, with extracellular antigen immunoreaction near depleted BALT (Supporting Information 6: Figure S5A,B). In the heart, p72‐positive macrophages were mainly perivascular, adjacent to hemorrhagic areas, and occasionally interspersed among myocardial fibers (Supporting Information 6: Figure S5C,D). Mild p72 immunoreaction was occasionally present in macrophages between diaphragm muscle fibers.

In the liver, p72 was detected at 7 dpi in monocytes and Kupffer cells within sinusoids (Figure 9E), and by 10 dpi, it was widespread in hepatocytes, macrophages, and extracellular deposits near mononuclear inflammation in portal and interlobular areas (Figure 9F; Supporting Information 7: Figure S6A,B). At this stage, moderate labeling was also detected in gallbladder mucosal macrophages near hemorrhagic areas (Supporting Information 7: Figure S6C,D). Scattered p72‐positive macrophages were found in the lamina propria of the stomach (Supporting Information 8: Figure S7A,B), small intestine (Supporting Information 9: Figure S8A,B), and large intestine (Supporting Information 9: Figure S8C,D), being slightly more numerous in the ileum. Occasional labeled macrophages were also noted in the pancreas adjacent to perivascular inflammation (Supporting Information 8: Figure S7C,D).

At 7 dpi, mild immunolabeling in the kidney was restricted to monocytes within glomerular capillaries and mesangial cells (Figure 9G). By 10 dpi, p72‐positive cells were observed in epithelial tubular cells and macrophages associated with interstitial nephritis and hemorrhages in cortex, medulla, and pelvis (Figure 9H; Supporting Information 10: Figure S9A,B). Rarely, p72‐positive macrophages were observed in the adrenal glands (Supporting Information 10: Figure S9C,D) and urinary bladder mucosa adjacent to hemorrhagic areas (Supporting Information 11: Figure S10A). Occasional macrophage labeling was also found in the synovial membrane (Supporting Information 11: Figure S10B). In the brain, mild to moderate immunostaining was detected in perivascular macrophages, especially in the choroid plexus, and occasionally in the neuroparenchyma (Supporting Information 11: Figure S10C,D). A significant positive correlation between IHCS and HS values was found (*ρ* = 0.943, *p*  < 0.01).

Animals from the negative control group rarely showed nonspecific diffuse immunolabeling in macrophages, lymphocytes, and plasma cells in various lymphoid tissues (palatine tonsils, spleen, bone marrow, lymph nodes) and the gastrointestinal mucosa, not associated with lesions. Immunohistochemical analysis of the HVI group with the IgG (isotype control) showed no significant immunolabeling (Supporting Information 12: Figure S11). Statistical analysis revealed significant differences in IHCS values among groups (K–W test: *p*  < 0.001). M‐W tests confirmed differences between the HVI group and both LVI and negative control groups (*p*  < 0.01), as well as between HVI and LVI–HVI groups (*p*  < 0.01) and between both LVI–HVI subgroups (*p*  < 0.05), whereas no differences were found between the LVI and negative control groups. IHCS values and significant differences among groups are shown in Figure 6B. Table 1 shows a comparative summary of ASFV p72 immunohistochemical antigen detection.

### 3.5. ASFV Isolation in PBMC Culture

ASFV isolation was assessed using an HAd assay, and viral load was quantified by qPCR at the third passage. In the LVI group, most samples were negative, with only low‐level viral activity (mutant isolate) detected in a few tissues (submandibular lymph nodes, palatine tonsils, kidneys) in case nos. 3 and 4 (HAd‐negative). In the LVI–HVI1 subgroup, detection of the Arm07 isolate with positive HAd was more frequent, particularly in case nos. 7, 10, and 11, with 50%–90% of tissues testing positive, mainly lymph nodes and palatine tonsils, occasionally including lungs, heart, and liver. The LVI–HVI2 subgroup showed restricted viral detection, primarily in lymph nodes (submandibular, mesenteric, gastrohepatic) of case nos. 14 and 15. Arm07 was detected in 25% of tissues in both cases (HAd‐positive in case No. 14, Had‐negative in case No. 15), with additional detection of the mutant isolate in 12.5%–25% of tissues. By contrast, the HVI group consistently showed high viral loads, with the Arm07 isolate detected by HAd in all examined tissues.

## 4. Discussion

Histopathological evaluation is essential for assessing lesion severity induced by ASFV isolates and provides critical insights into tissue alterations caused by vaccination or infection [42, 43]. These analyses allow detection of subtle changes not evident from clinical or molecular assessments alone, offering a comprehensive understanding of vaccine‐associated effects. However, histopathological findings can be influenced by factors such as secondary infections, trauma, or underlying conditions affecting the immune system [29]. Therefore, histopathology must be complemented with IHC to directly correlate observed lesions with viral presence. The main aim of this study is to employ these diagnostic tools to assess the safety of the vaccine prototype (Lv17/WB/Rie1‐*Δ*CD), reporting significant differences between the different vaccination procedures performed.

Most histopathological and IHC studies of ASF in wild boar have focused on moderate or highly virulent isolates [30–33], leaving a gap in our understanding of the tissue‐level effects caused by low virulence strains in this species. This work provides, for the first time, a comprehensive histological and immunohistochemical description of a low virulent ASFV strain in wild boar. When comparing the evolution of lesions in animals exposed to Lv17/WB/Rie1‐*Δ*CD isolate in the different groups, it became evident that the low virulence strain caused only limited and transient tissue alterations, especially in the lymphoid tissue. Animals exclusively exposed to the LVI (without challenge) presented minimal lymphoid depletion and mitosis in the germinal center, suggesting a prior mild lymphocytolysis, particularly in lymph nodes [39]. IHC revealed only scarce and low‐intensity antigen immunostaining in specific lymph nodes and spleen. Nevertheless, this cytoplasmic immunostaining showed no inclusion bodies associated with viral replication factories. In many cases, it was not correlated with existing lesions; therefore, it most likely represents residual viral antigen, although nonspecific background staining cannot be fully excluded. This is consistent with the low viral loads and the small number of tissues that tested positive for the mutant isolate, indicating that viral clearance progresses over time, becoming more evident at later stages (30 dpv).

Viral antigen immunoreaction in the palatine tonsils was slightly more frequent and was associated with minimal epithelial necrosis and mild mononuclear infiltration within the tonsillar crypts. This aligns with viral shedding studies, which reported that wild boars infected with Lv17/WB/Rie1 isolate exhibited a prolonged shedding period in oral fluid [44]. This suggests that orally infected animals with an attenuated isolate may develop limited viral persistence at the entry site, particularly in the tonsils and adjacent lymph nodes [45], but with effective containment of infection at mucosal barriers.

Previous studies have shown that Lv17/WB/Rie1 [46–48] and its derivative Lv17/WB/Rie1‐*Δ*CD (23) are capable of inducing partial protection against ASFV in domestic pigs [23, 46, 47]. In the present study, we extend these observations to wild boar, confirming that the orally inoculated Lv17/WB/Rie1‐*Δ*CD isolate exhibits reduced virulence compared to the parental Lv17/WB/Rie1, already analyzed in our previous experiments [34, 35]. Lv17/WB/Rie1 isolate occasionally presented higher viral loads, more pronounced lymphoid depletion, and mild mononuclear inflammatory infiltrates [39]. This finding aligns with previous observations in domestic pigs, where parenteral administration of Lv17/WB/Rie1 induced stronger inflammatory responses and transient hematological changes [46].

The study of LVI–HVI group (vaccinated and challenged animals) revealed interesting insights into viral distribution and lesion development. The LVI‐HVI1 subgroup had a higher viral load compared with LVI–HVI2 subgroup, with a moderate percentage of tissues testing positive for the Arm07 isolate; however, most tissues displayed mild or no lesions, indicating effective partial control of viral replication. By contrast, LVI–HVI2 subgroup only had sporadic positivity, mainly confined to a few number of lymph nodes, for both Arm07 and Lv17/WB/Rie1‐*Δ*CD isolates, with notably reduced viral persistence compared to single‐dose vaccination. This aligns with the IHC analysis, which demonstrates specific antigenic immunostaining exclusively in LVI–HVI1 macrophages with inclusion bodies, directly correlated with mild lymphocytolysis and TBM infiltration in the palatine tonsils and some lymph nodes. This suggests that single‐dose vaccination, though capable of controlling the virus, may benefit from booster doses to enhance viral clearance and further limit the occurrence of transient mild lesions in the lymphoid system.

Both subgroups exhibited a mortality of 16.7 % (one out of six animals), with case No. 8 from LVI–HVI1 not satisfactorily immunized, showing characteristic virus antigen dissemination and lesions of acute ASF. In contrast, the negative immunostaining in lesions of case No. 17 ruled out direct virus‐induced damage, suggesting successful immunization. Aggressive interactions causing high stress and subsequent immunosuppression [49], along with exposure to both isolates or opportunistic pathogens, may have contributed to the clinical outcome in the affected animal. The remaining revaccinated animals displayed well‐developed lymphoid tissue, supporting the idea that observed lesions were transient and nonprogressive. These changes reflect a temporary, prolonged viral persistence rather than chronic infection, ultimately leading to viral elimination [50] and regeneration of lymphoid cells over time.

Variations in dosage can significantly influence clinical and pathological outcomes [51], making dose optimization crucial to maintain vaccine safety and efficacy [52]. This underscores the relevance of revaccination protocols, particularly for oral administration, to enhance immunity and promote rapid viral clearance [34]. In summary, the gene‐deleted Lv17/WB/Rie1‐*Δ*CD candidate demonstrated a favorable safety profile and reduced pathogenicity, supporting its potential use in oral vaccination strategies. However, further studies are needed to assess long‐term genetic stability and the risk of reversion to virulence [23].

The HVI group (unvaccinated control pigs) developed acute ASF, displaying severe clinical signs, extensive viral dissemination, and intense lesions. Results indicated more pronounced inflammatory, hemorrhagic, and necrotic lesions at 10 dpi, associated with higher viral loads and antigen spread across various cell types. These findings are consistent with studies on moderate to highly virulent isolates, where infected cells progressively increase and eventually spread to other cell types, such as hepatocytes, tonsillar crypt epithelial cells, renal interstitial cells, or capillary endothelial cells [53, 54]. During the late acute phase, viral antigen was mildly detected in “secondary organs” such as the heart, adrenal gland, urinary bladder, gallbladder, gastrointestinal tract, and CNS, aligning with studies that reported this antigen location mainly in the subacute form [28]. These results do not fully correlate with qPCR data, where Ct values did not differ significantly between organs. This suggests that the viral loads detected in “secondary organs", with few components of the mononuclear phagocyte system, may be primarily attributed to the circulating monocytes and, to a lesser extent, to infected mononuclear infiltrates, which are more frequently extended in later stages.

The expression of p72 in macrophages containing inclusion bodies, together with the isolation results from the LVI–HVI1 subgroup, indicates that the observed necrotic lesions are a direct consequence of Arm07 infection, mediated by the secretion of TNF‐*α*, IL‐1, and IL‐6 [26, 55]. The virus was confined exclusively to resident macrophages in the subcapsular, trabecular, and medullary sinuses, suggesting effective containment. This may be linked to IFN‐*γ* responses from these macrophages [56], supporting the hypothesis that lymph nodes act as an innate immune “barrier” [57], preventing viral spread to vital organs and disease severity. This protective system may be enhanced by adaptive immunity induced through vaccination. Although some studies support a correlation between increased IFN‐*γ* secretion and protection against ASFV [58, 59], others have not found a direct relationship [48] or reported no significant increase in cytokine levels in immunized animals [46]. Further research on cellular immunity is required to clarify its role and potential immunopathological effects in vaccinated animals.

A limitation of this study is the use of different inoculation routes for the attenuated and virulent isolates, which may have influenced viral entry, dissemination, and lesion severity. This design was chosen to reproduce field‐relevant oral vaccination conditions while maintaining a standardized and reproducible model of acute ASFV infection for the challenge control. Future studies employing matched inoculation routes could help further distinguish the effects of viral virulence from those associated with the route of exposure.

## 5. Conclusion

Histopathology and IHC, and allowed the identification of key outcomes in ASFV infection and vaccination studies. All unvaccinated, IM‐infected animals (HVI) succumbed to fatal disease with high antigen loads. The LVI persisted mainly at the oral entry point, and changes observed following vaccination reflected a transient, not chronic, infection. Single‐dose vaccination (LVI–HVI1) provided partial protection, whereas repeated oral vaccination (LVI–HVI2) markedly reduced viral antigen burden and mortality after Arm07 challenge. These results underscore the value of such analyses for understanding ASF pathogenesis, tissue damage, viral distribution, and immune responses. The application of these tools should be recommended in multidisciplinary research to evaluate vaccine candidates and protocols, emphasizing the central role of the lymphoid system in immune defense. Further investigation into viral persistence and pathogenic mechanisms is needed to clarify potential vaccine‐induced effects and long‐term protection, which will be essential to refine vaccination protocols and improve ASF management.

## Conflicts of Interest

The authors declare no conflicts of interest.

## Funding

This research was funded by the Research Project (Grant CPP2023‐010867), financed by MICIU/AEI/10.13039/501100011033 and by FEDER, EU. Jose Ángel Barasona is a recipient of a “Ramón y Cajal” contract (Grant RYC2022‐038060‐I) funded by the Spanish Ministry of Science and Innovation (MCIN/AEI) and Fondo Social Europeo Plus (FSE+).

## Supporting Information

Additional supporting information can be found online in the Supporting Information section.

## Supporting information


**Supporting Information 1** Table S1: Individual animal data: group, timepoints, histopathology and immunohistochemistry scores, ASFV blood Ct values and ASFV isolation.

**Supporting Information 2** Figure S1: Immunohistochemical p72 ASFV detection in palatine tonsils and thymus of HVI‐infected wild boars.

**Supporting Information 3** Figure S2: Immunohistochemical p72 ASFV detection in bone marrow and spleen of HVI‐infected wild boars.

**Supporting Information 4** Figure S3: Immunohistochemical p72 ASFV detection in submandibular, ileocecal, gastrohepatic, and renal lymph nodes of HVI‐infected wild boars.

**Supporting Information 5** Figure S4: Immunohistochemical p72 ASFV detection in inguinal, retropharyngeal, mediastinal, and mesenteric lymph nodes of HVI‐infected wild boars.

**Supporting Information 6** Figure S5: Immunohistochemical p72 ASFV detection in lungs and heart of HVI‐infected wild boars.

**Supporting Information 7** Figure S6: Immunohistochemical p72 ASFV detection in liver and gallbladder of HVI‐infected wild boars.

**Supporting Information 8** Figure S7: Immunohistochemical p72 ASFV detection in stomach and pancreas of HVI‐infected wild boars.

**Supporting Information 9** Figure S8: Immunohistochemical p72 ASFV detection in small and large intestine of HVI‐infected wild boars.

**Supporting Information 10** Figure S9: Immunohistochemical p72 ASFV detection in kidneys and adrenal glands of HVI‐infected wild boars.

**Supporting Information 11** Figure S10: Immunohistochemical p72 ASFV detection in urinary bladder, synovial membrane and brain of HVI‐infected wild boars.

**Supporting Information 12** Figure S11: Negative immunohistochemical controls for ASFV p72 detection in HVI and control (healthy) wild boars.

## Data Availability

The data used to support the findings of this study are included within the article and supplemental files.
