# Supplementary material for: Viral Distribution of Wild Boar Exposed to Low (Vaccine Candidate) and High Virulence African Swine Fever Virus Isolates: Immunohistochemical Characterization
Source: Transbound Emerg Dis. 2025 Dec 9;2025:4258247. doi: 10.1155/tbed/4258247 (PMC12697812; doi:10.1155/tbed/4258247)
Supplement: Supplementary file 1 — Supporting Information 1 Table S1: Individual animal data: group, timepoints, histopathology and immunohistochemistry scores, ASFV blood Ct values and ASFV isolation. [file TBED-2025-4258247-s001.docx]

**Supporting information**

**Table S1.** Details of each animal according to the group, days post-vaccination, days post-callenge or days post-infection/inoculation, histopathologic score, immunohistochemical score, African swine fever virus Ct values (blood) and isolation.

| **Case No.** | **Group** | **DPV** | **DPC/DPI** | **HS** | **IHCS** | **ASFV Ct value (blood)** | **ASFV mean Ct value (tissues)** | **ASFV isolation (% positive tissues)**** | | |
| --- | --- | --- | --- | --- | --- | --- | --- | --- | --- | --- |
|  |  |  |  |  |  |  |  | **Arm07** | **Lv17/WB/Rie1-ΔCD** | **Negative** |
| 1 | LVI | 30 | - | 21 | 0 | 32.50 | 39.09 | 0 | 0 | 100 |
| 2 |  | 30 | - | 15 | 0 | 39.45 | 39.54 | 0 | 0 | 100 |
| 3 |  | 30 | - | 27 | 5 | 36.14 | 37.90 | 0 | 50 | 50 |
| 4 |  | 30 | - | 23 | 1* | 3.40 | 38.33 | 0 | 100 | 0 |
| 5 |  | 30 | - | 25 | 4 | 33.06 | 37.66 | 0 | 50 | 50 |
| 6 |  | 30 | - | 12 | 0 | 40 | 39.75 | 0 | 0 | 100 |
| 7 | LVI-HVI1 | 30 | 30 | 31 | 3 | 24.09 | 34.37 | 90 | 0 | 10 |
| 8 |  | 30 | 30 | 72 | 33 | 17.41 | 20.93 | 100 | 0 | 0 |
| 9 |  | 30 | 30 | 29 | 2 | 25.65 | 35.03 | 50 | 0 | 50 |
| 10 |  | 30 | 30 | 45 | 12 | 26.10 | 32.38 | 87.5 | 0 | 12.5 |
| 11 |  | 30 | 30 | 18 | 1* | 33.06 | 38.26 | 75 | 0 | 25 |
| 12 |  | 30 | 30 | 23 | 2* | 35.36 | 38.18 | 18 | 18 | 64 |
| 13 | LVI-HVI2 | 30/14 | 30 | 18 | 0 | 40 | 38.61 | 0 | 25 | 75 |
| 14 |  | 30/14 | 30 | 22 | 1* | 32.57 | 35.87 | 25 | 12.5 | 62.5 |
| 15 |  | 30/14 | 30 | 17 | 0 | 40 | 38.78 | 25 | 25 | 50 |
| 16 |  | 30/14 | 30 | 10 | 0 | 40 | 40.00 | 0 | 0 | 100 |
| 17 |  | 30/14 | 30 | 53 | 2* | 40 | 35.93 | 0 | 0 | 100 |
| 18 |  | 30/14 | 30 | 14 | 0 | 40 | 39.91 | 0 | 0 | 100 |
| 19 | HVI | - | 7 | 89 | 35 | 19.30 | 21.25 | 100 | 0 | 0 |
| 20 |  | - | 7 | 108 | 43 | 22.20 | 19.58 | 100 | 0 | 0 |
| 21 |  | - | 7 | 76 | 34 | 21.99 | 20.41 | 54.6 | 0 | 45.4 |
| 22 |  | - | 10 | 117 | 53 | 16.71 | 23.20 | 100 | 0 | 0 |
| 23 |  | - | 10 | 132 | 58 | 13.90 | 18.97 | 100 | 0 | 0 |
| 24 |  | - | 10 | 118 | 50 | 14.68 | 25.99 | 100 | 0 | 0 |
| 25 | Control | - | - | 8 | 0 | 40 | 40.00 | - | - | - |
| 26 |  | - | - | 9 | 0 | 40 | 40.00 | - | - | - |
| 27 |  | - | - | 12 | 0 | 40 | 40.00 | - | - | - |
| 28 |  | - | - | 11 | 0 | 40 | 40.00 | - | - | - |
| 29 |  | - | - | 8 | 0 | 40 | 40.00 | - | - | - |
| 30 |  | - | - | 10 | 0 | 40 | 40.00 | - | - | - |

* The ASFV immunoexpression was not related with any adjacent lesion.

** Percentage of tissues positive for ASFV isolation in PBMC culture, calculated from samples previously confirmed as qPCR-positive.

Abbreviations: HVI, high virulent isolate; LVI, low virulent isolate; LVI-HVI, low virulent – high virulent isolate; DPV, days post-vaccination; DPC, days post-callenge; DPI, days post-infection/inoculation; HS, histopathologic score; IHCS, immunohistochemical score; ASFV, African swine fever virus.
