# Supplementary figures and images for: Viral Distribution of Wild Boar Exposed to Low (Vaccine Candidate) and High Virulence African Swine Fever Virus Isolates: Immunohistochemical Characterization
Source: Transbound Emerg Dis. 2025 Dec 9;2025:4258247. doi: 10.1155/tbed/4258247 (PMC12697812; doi:10.1155/tbed/4258247)

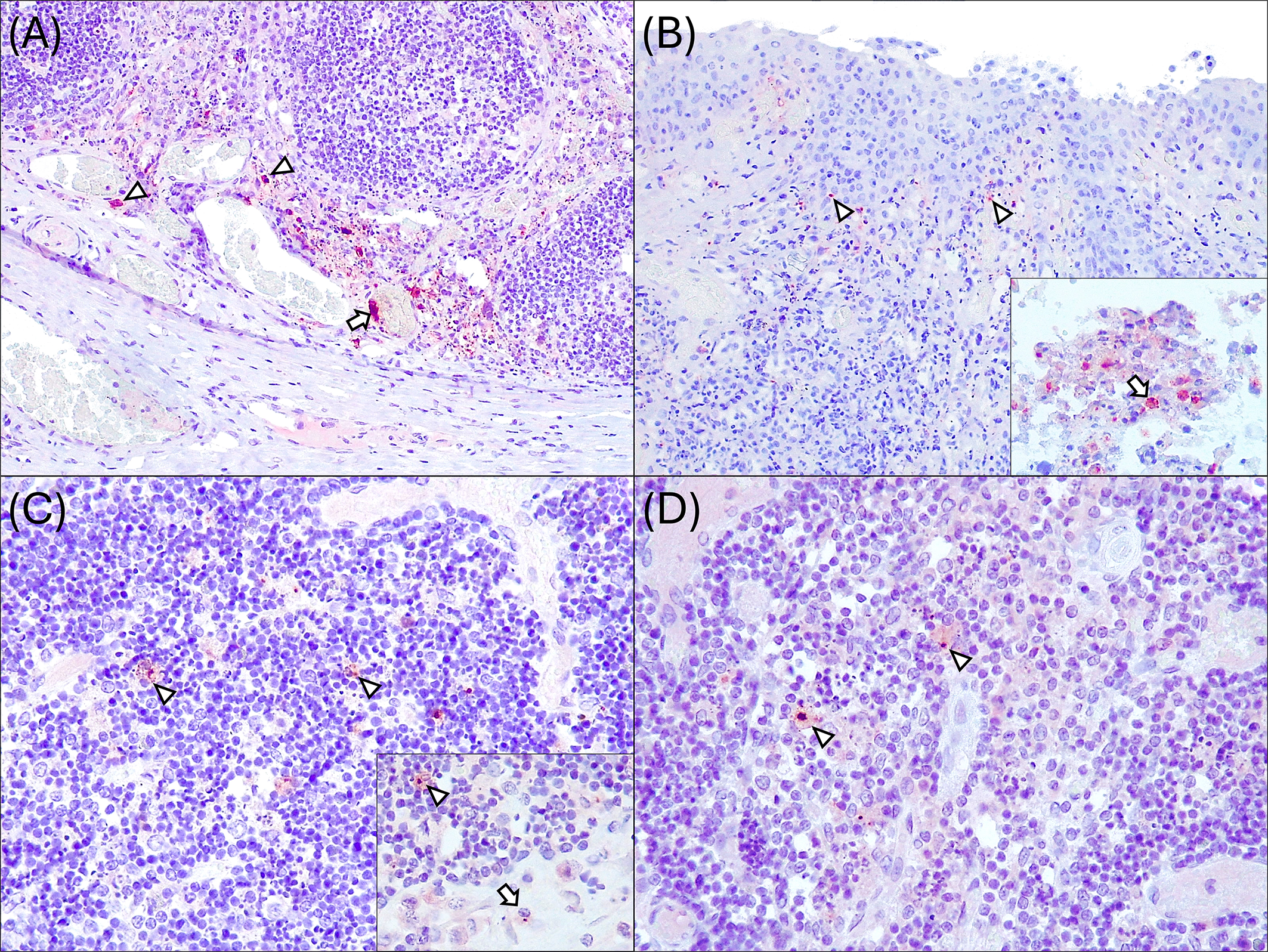

Supplement: Supplementary file 2 — Supporting Information 2 Figure S1: Immunohistochemical p72 ASFV detection in palatine tonsils and thymus of HVI‐infected wild boars. [file TBED-2025-4258247-s002.tif]

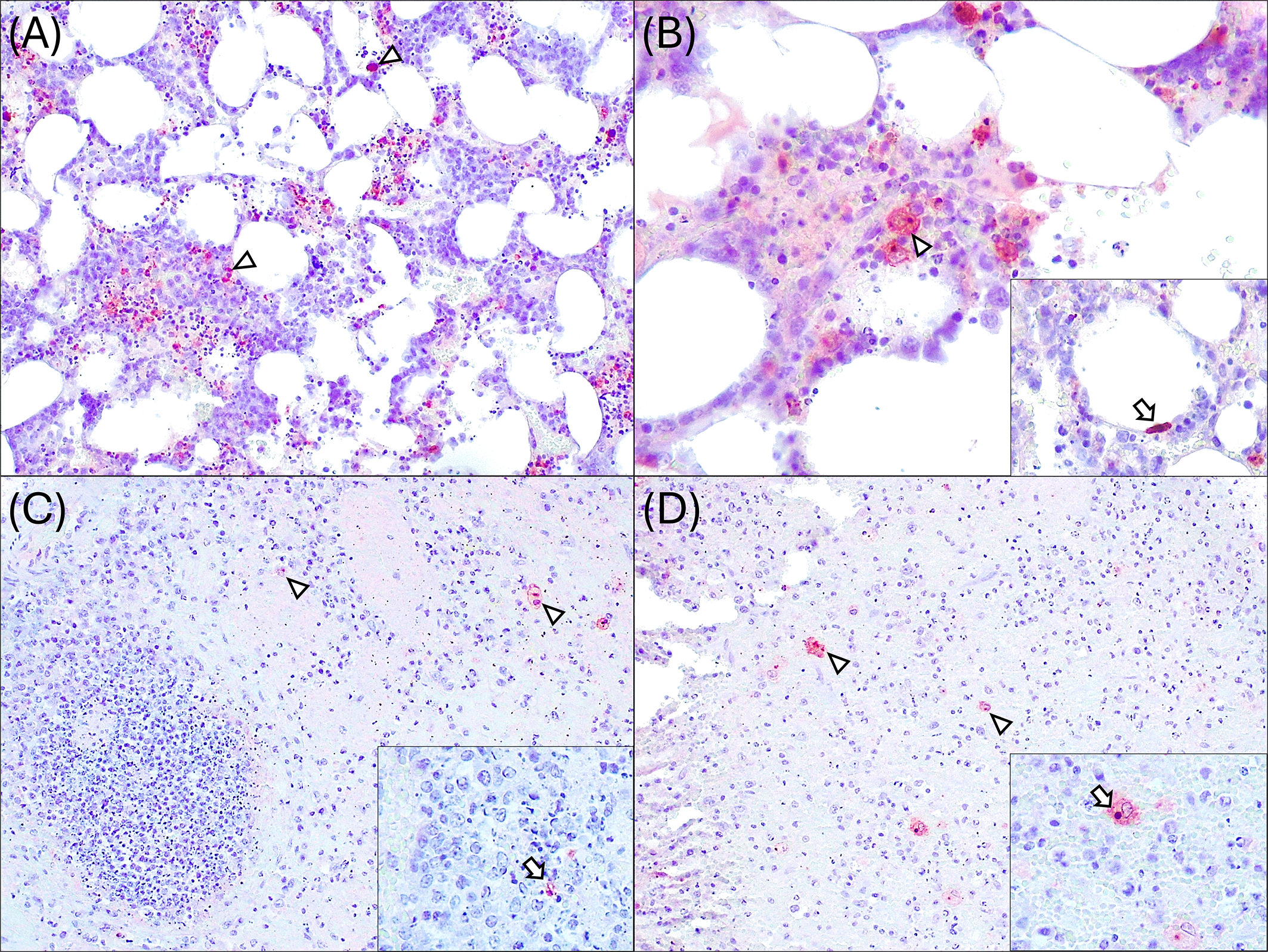

Supplement: Supplementary file 3 — Supporting Information 3 Figure S2: Immunohistochemical p72 ASFV detection in bone marrow and spleen of HVI‐infected wild boars. [file TBED-2025-4258247-s003.tif]

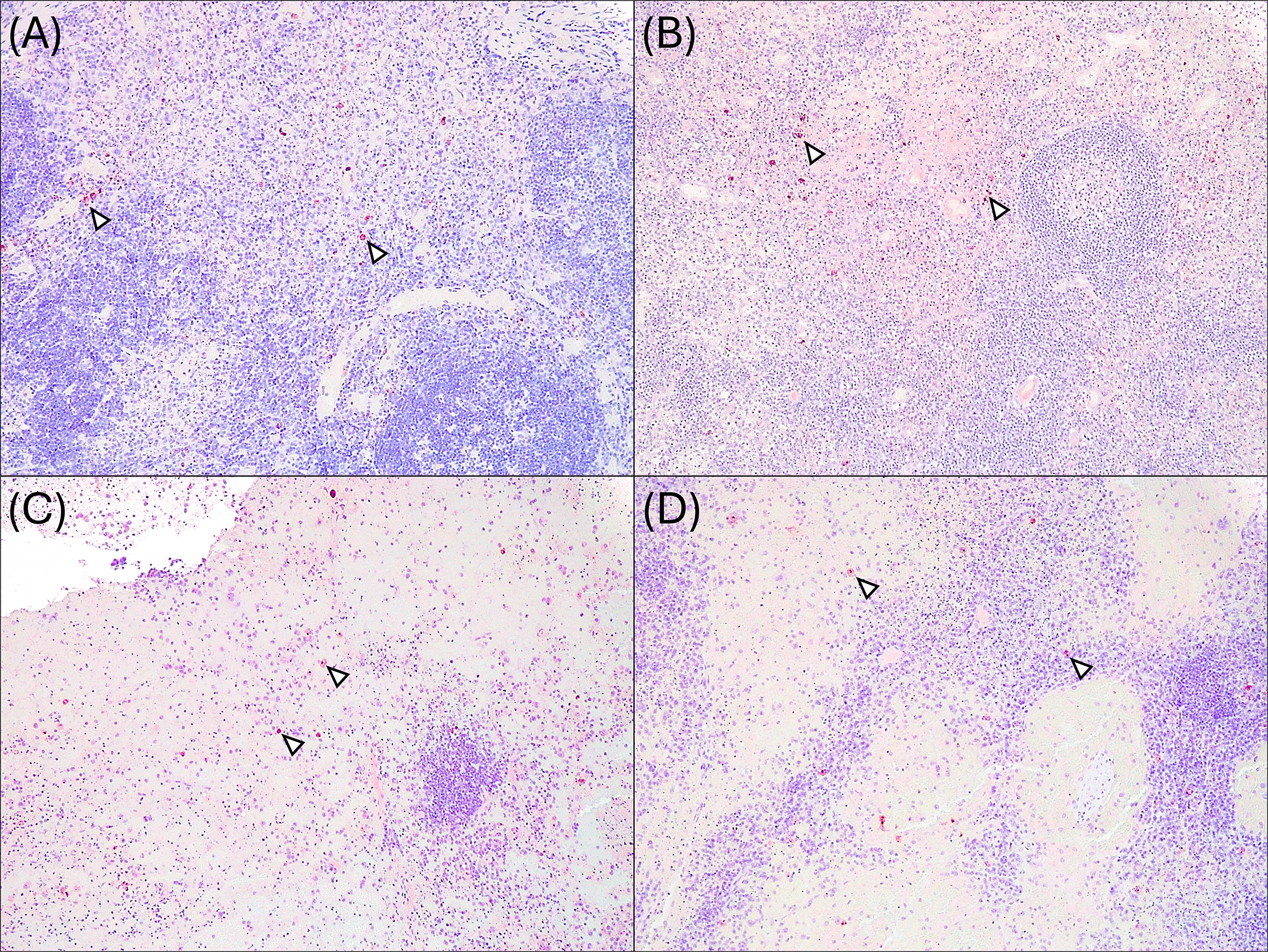

Supplement: Supplementary file 4 — Supporting Information 4 Figure S3: Immunohistochemical p72 ASFV detection in submandibular, ileocecal, gastrohepatic, and renal lymph nodes of HVI‐infected wild boars. [file TBED-2025-4258247-s004.tif]

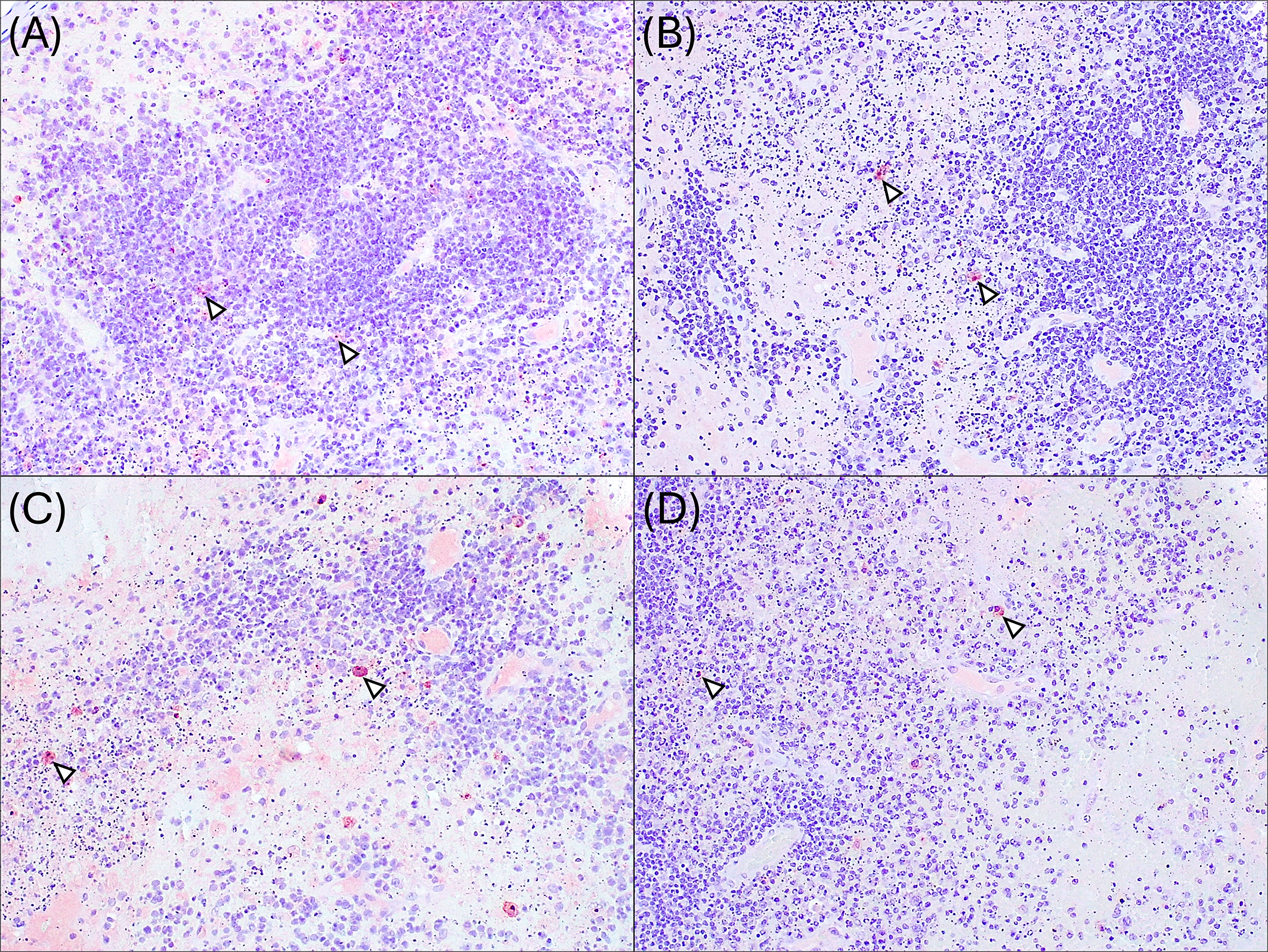

Supplement: Supplementary file 5 — Supporting Information 5 Figure S4: Immunohistochemical p72 ASFV detection in inguinal, retropharyngeal, mediastinal, and mesenteric lymph nodes of HVI‐infected wild boars. [file TBED-2025-4258247-s005.tif]

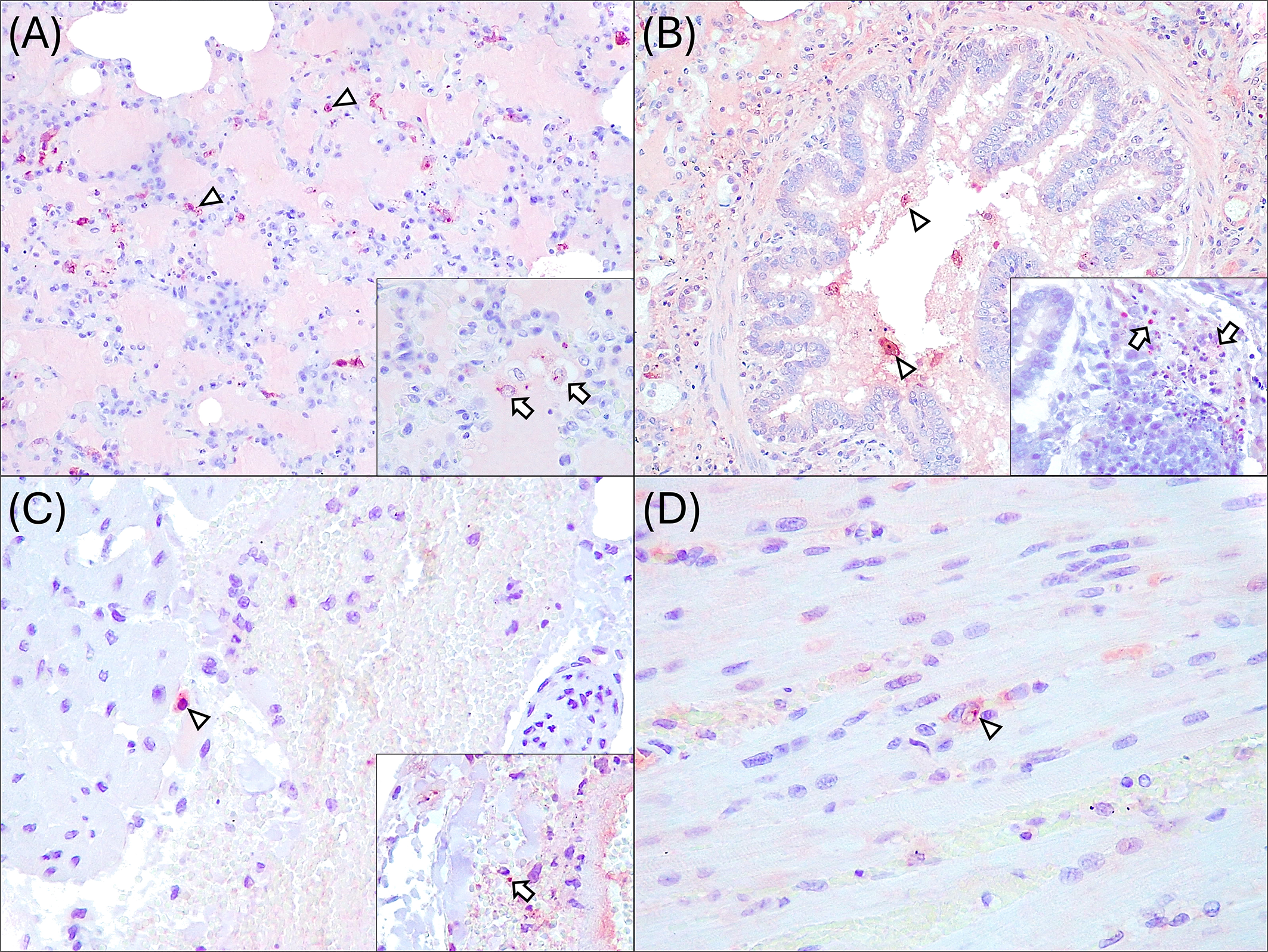

Supplement: Supplementary file 6 — Supporting Information 6 Figure S5: Immunohistochemical p72 ASFV detection in lungs and heart of HVI‐infected wild boars. [file TBED-2025-4258247-s007.tif]

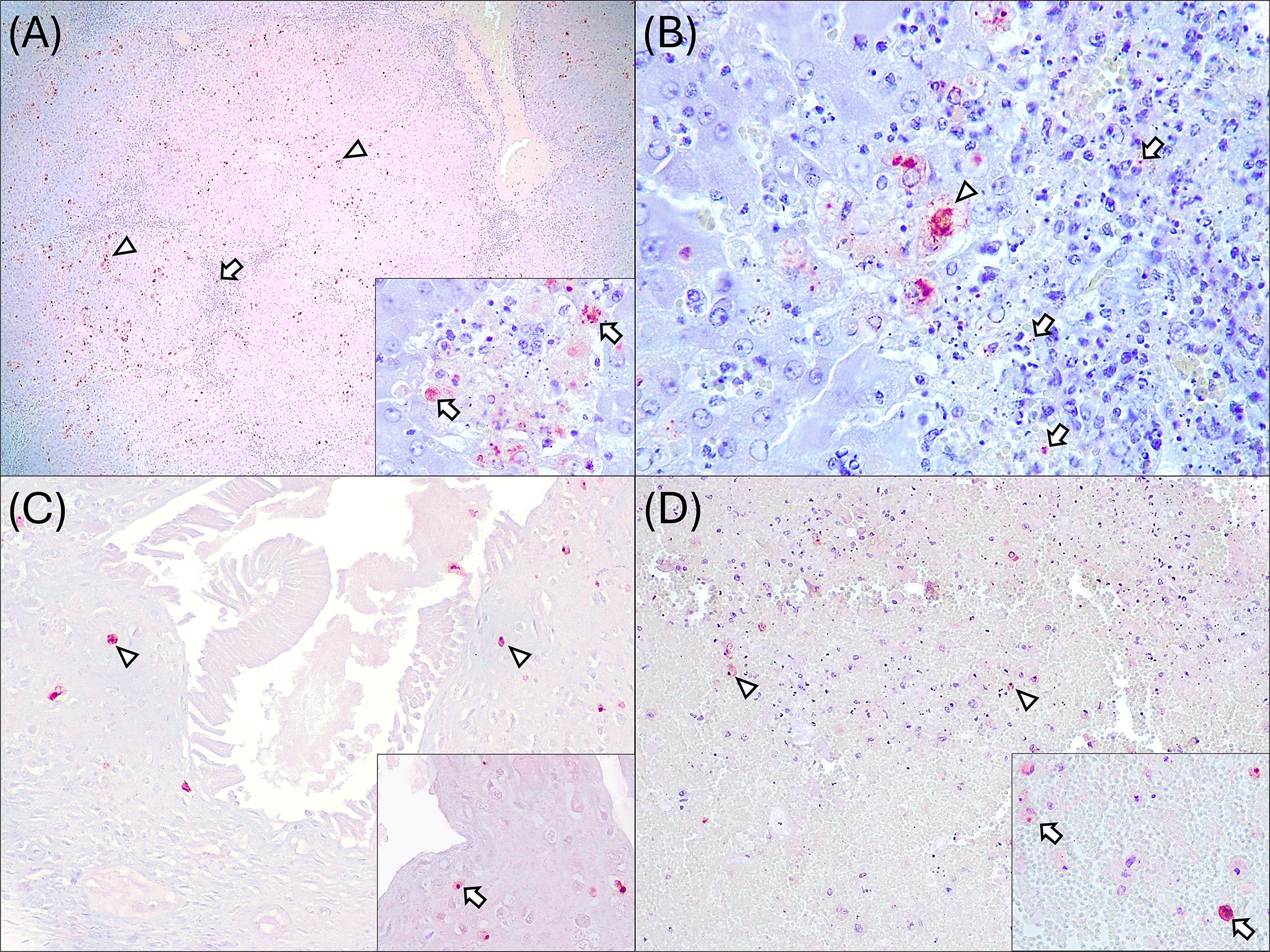

Supplement: Supplementary file 7 — Supporting Information 7 Figure S6: Immunohistochemical p72 ASFV detection in liver and gallbladder of HVI‐infected wild boars. [file TBED-2025-4258247-s008.tif]

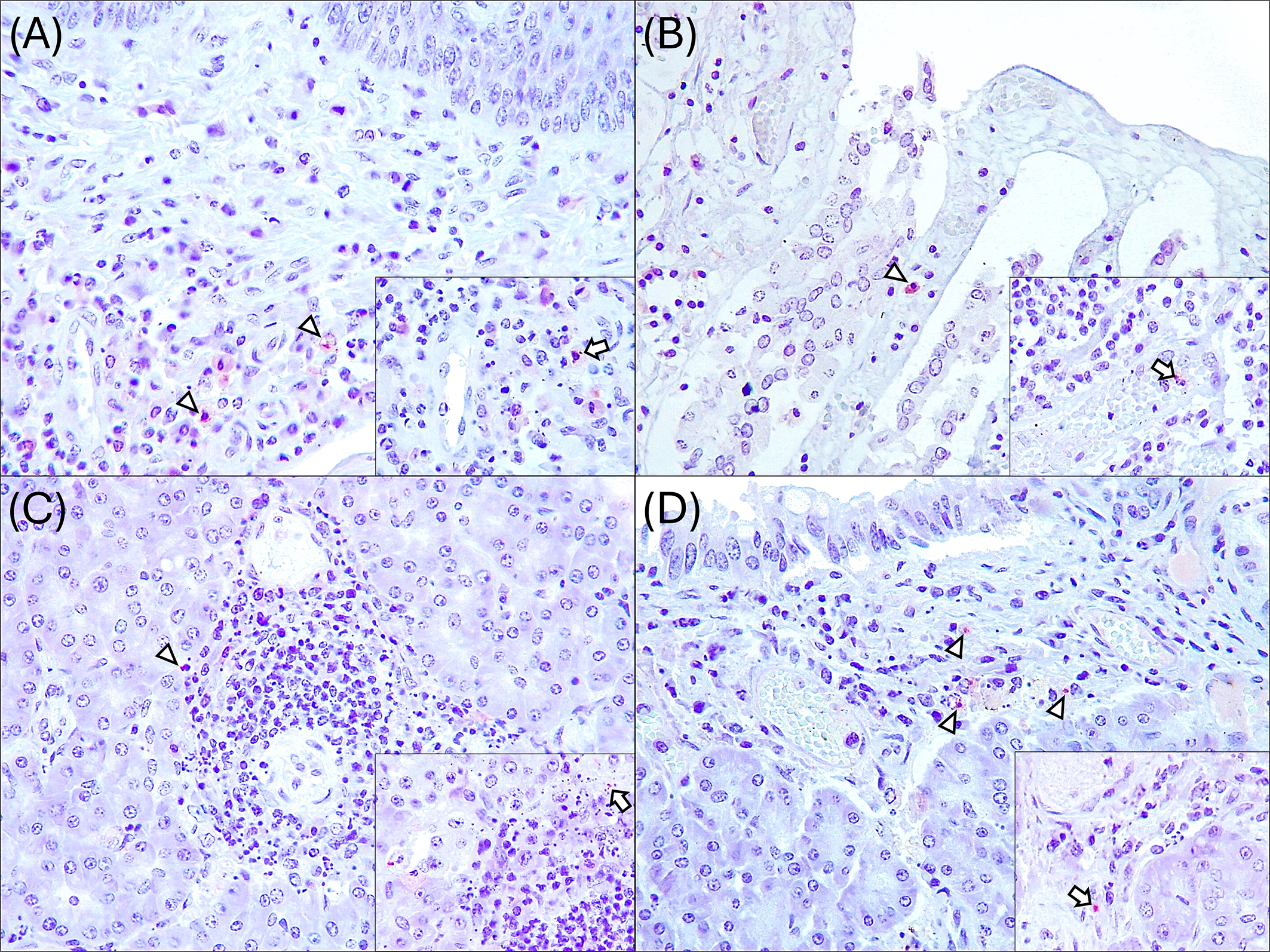

Supplement: Supplementary file 8 — Supporting Information 8 Figure S7: Immunohistochemical p72 ASFV detection in stomach and pancreas of HVI‐infected wild boars. [file TBED-2025-4258247-s009.tif]

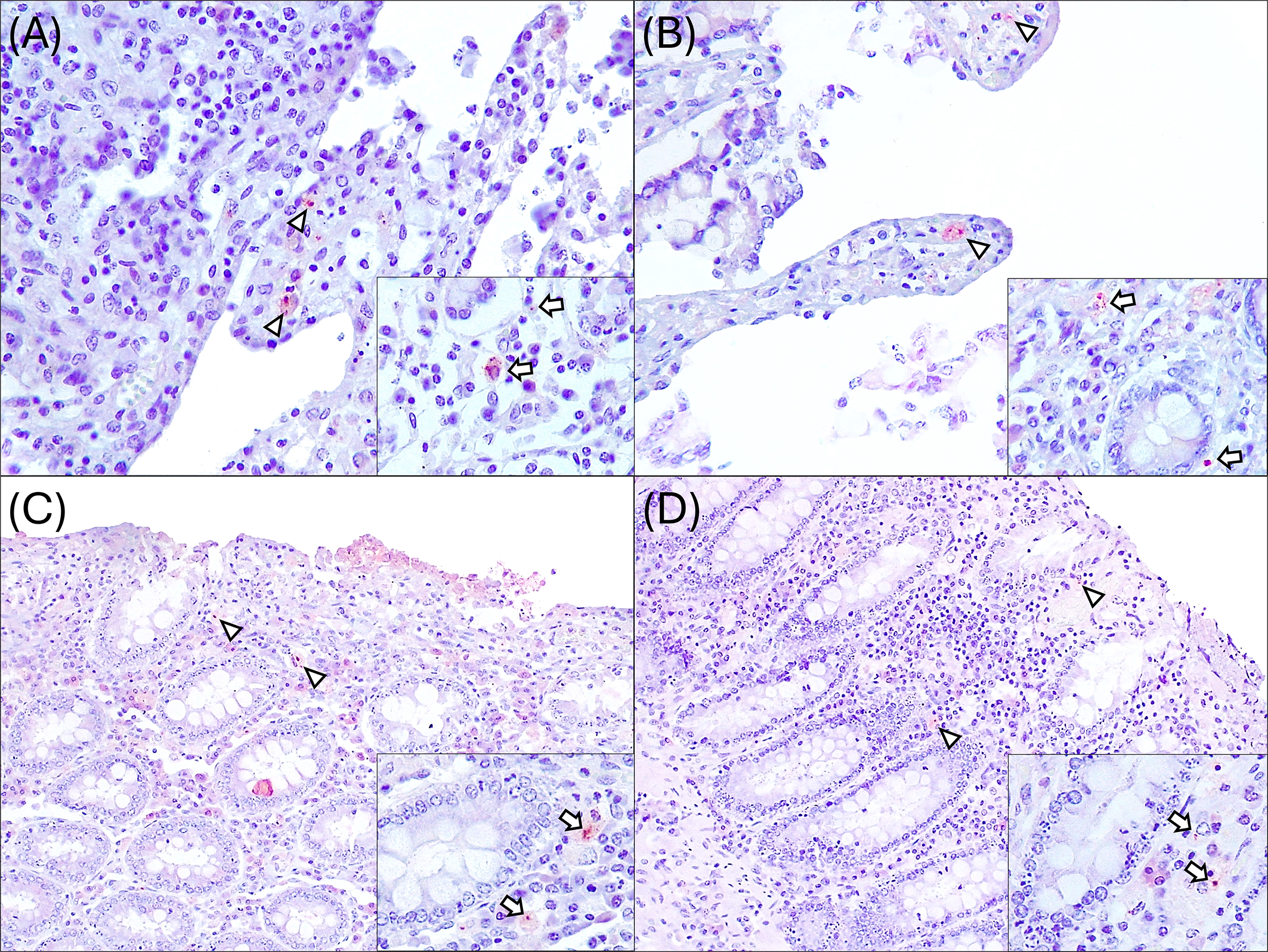

Supplement: Supplementary file 9 — Supporting Information 9 Figure S8: Immunohistochemical p72 ASFV detection in small and large intestine of HVI‐infected wild boars. [file TBED-2025-4258247-s012.tif]

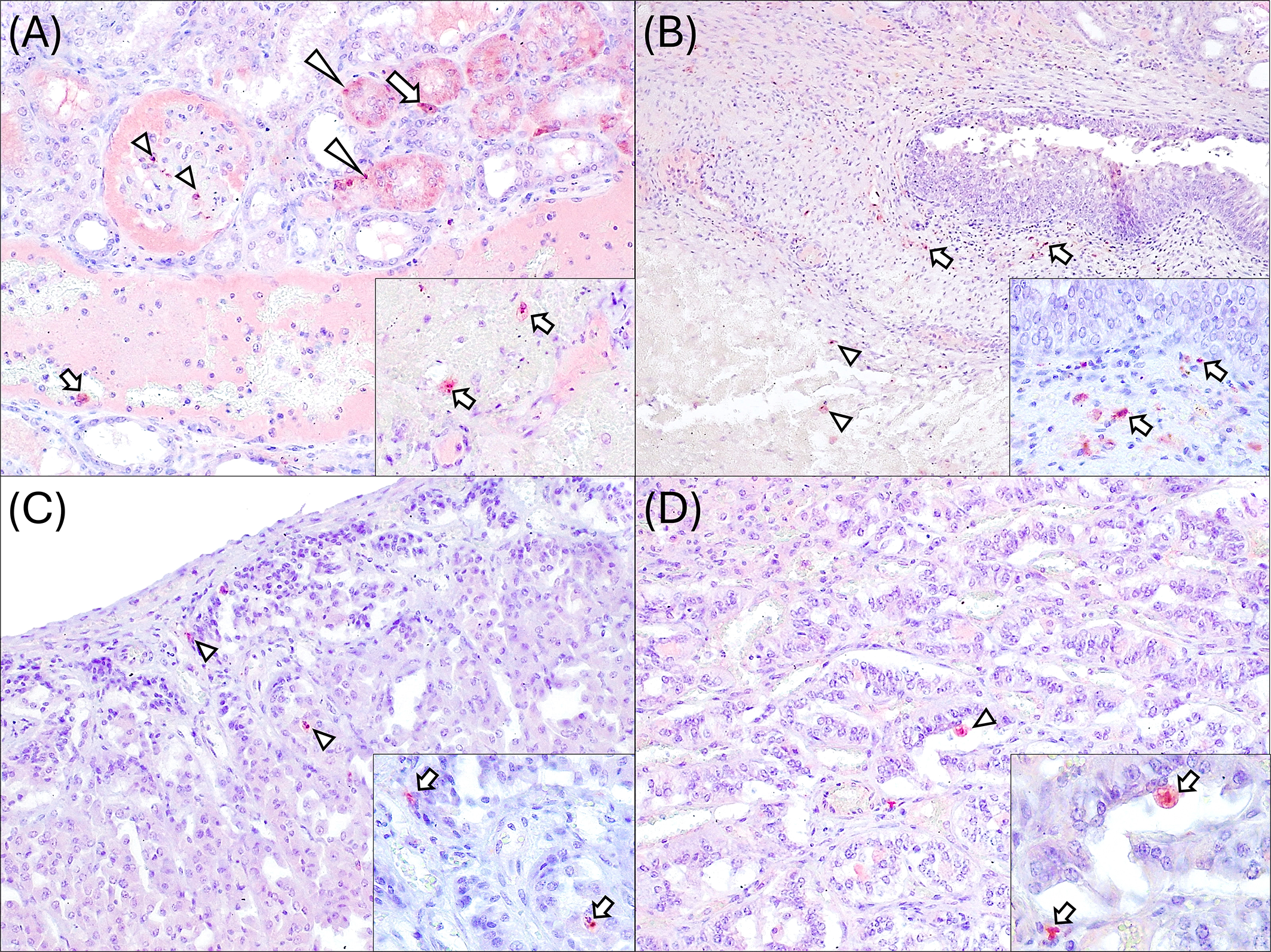

Supplement: Supplementary file 10 — Supporting Information 10 Figure S9: Immunohistochemical p72 ASFV detection in kidneys and adrenal glands of HVI‐infected wild boars. [file TBED-2025-4258247-s010.tif]

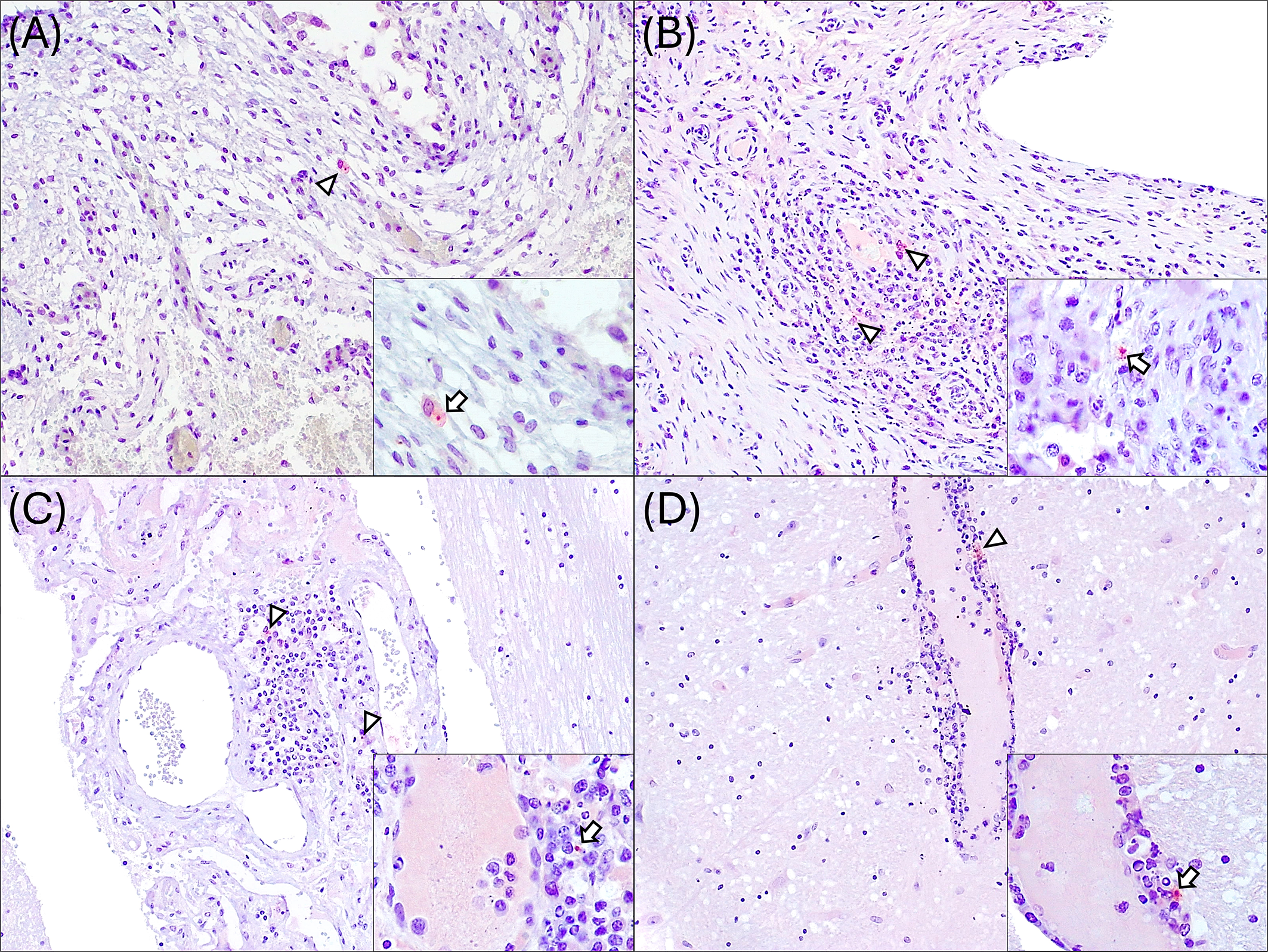

Supplement: Supplementary file 11 — Supporting Information 11 Figure S10: Immunohistochemical p72 ASFV detection in urinary bladder, synovial membrane and brain of HVI‐infected wild boars. [file TBED-2025-4258247-s011.tif]

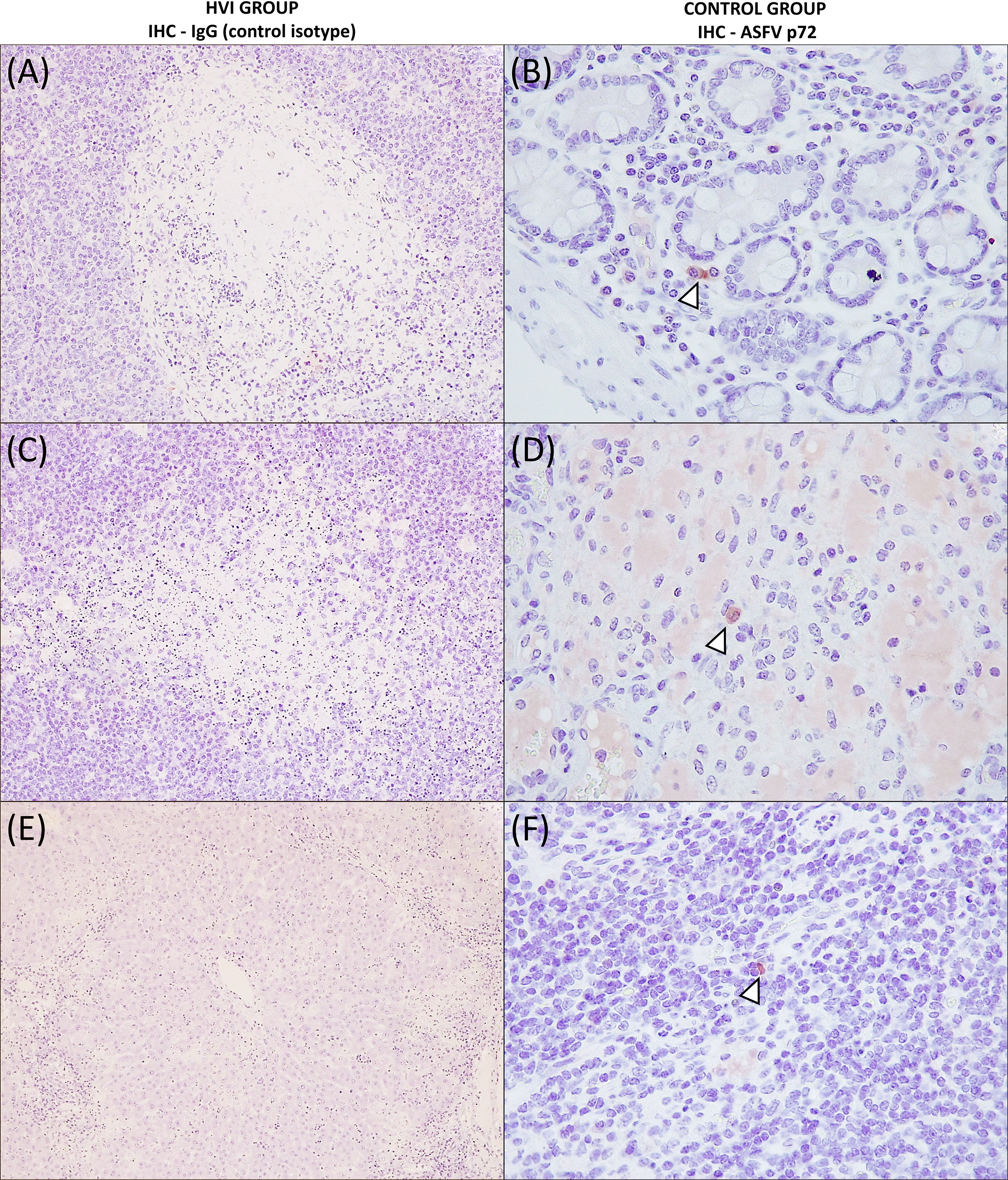

Supplement: Supplementary file 12 — Supporting Information 12 Figure S11: Negative immunohistochemical controls for ASFV p72 detection in HVI and control (healthy) wild boars. [file TBED-2025-4258247-s006.tif]
